# The Fragile X Protein binds mRNAs involved in cancer progression and modulates metastasis formation

**DOI:** 10.1002/emmm.201302847

**Published:** 2013-09-16

**Authors:** Rossella Lucá, Michele Averna, Francesca Zalfa, Manuela Vecchi, Fabrizio Bianchi, Giorgio La Fata, Franca Del Nonno, Roberta Nardacci, Marco Bianchi, Paolo Nuciforo, Sebastian Munck, Paola Parrella, Rute Moura, Emanuela Signori, Robert Alston, Anna Kuchnio, Maria Giulia Farace, Vito Michele Fazio, Mauro Piacentini, Bart De Strooper, Tilmann Achsel, Giovanni Neri, Patrick Neven, D Gareth Evans, Peter Carmeliet, Massimiliano Mazzone, Claudia Bagni

**Affiliations:** 1 VIB Center for the Biology of DiseaseLeuven, Belgium; 2 Center for Human GeneticsKU Leuven, Belgium; 3 CIR Department, Faculty of Medicine, University “Campus Bio-Medico”Rome, Italy; 4 Department of Biomedicine and Prevention, University “Tor Vergata”Rome, Italy; 5 IFOM, Fondazione Istituto FIRC di Oncologia MoleculareMilan, Italy; 6 Molecular Medicine Program, Department of Experimental Oncology, European Institute of OncologyMilan, Italy; 7 National Institute for Infectious Diseases, IRCCS ‘Lazzaro Spallanzani’Rome, Italy; 8 Vall d'Hebron Institute of Oncology, Vall d'Hebron University HospitalBarcelona, Spain; 9 Laboratory of Oncology, IRCCS H. “Casa Sollievo della Sofferenza”San Giovanni Rotondo, Italy; 10 Institute of Translational Pharmacology, CNRRome, Italy; 11 Cancer Research UK Paediatric and Familial Cancer Research Group, Manchester Academic Health Science Centre, University of ManchesterManchester, UK; 12 Laboratory of Angiogenesis and Neurovascular Link, Vesalius Research Center, VIBLeuven, Belgium; 13 Laboratory of Angiogenesis and Neurovascular Link, Department of OncologyKU Leuven, Belgium; 14 University Campus “Bio-Medico”, Section of Molecular Medicine and BiotechnologyRome, Italy; 15 Department of Biology, University “Tor Vergata”Rome, Italy; 16 Institute of Medical Genetics, Catholic UniversityRome, Italy; 17 Department of Obstetrics and Gynaecology, University Hospitals LeuvenLeuven, Belgium; 18 Genomic Medicine, Manchester Academic Health Science Centre, Central Manchester Foundation Trust, St. Mary's HospitalManchester, UK; 19 Laboratory of Molecular Oncology and Angiogenesis, Vesalius Research Center, VIBLeuven, Belgium; 20 Laboratory of Molecular Oncology and Angiogenesis, Department of OncologyKU Leuven, Belgium

**Keywords:** cell invasion, EMT, FMRP, mRNA metabolism, TNBC

## Abstract

The role of the fragile X mental retardation protein (FMRP) is well established in brain, where its absence leads to the fragile X syndrome (FXS). FMRP is almost ubiquitously expressed, suggesting that, in addition to its effects in brain, it may have fundamental roles in other organs. There is evidence that FMRP expression can be linked to cancer. *FMR1* mRNA, encoding FMRP, is overexpressed in hepatocellular carcinoma cells. A decreased risk of cancer has been reported in patients with FXS while a patient-case with FXS showed an unusual decrease of tumour brain invasiveness. However, a role for FMRP in regulating cancer biology, if any, remains unknown. We show here that FMRP and *FMR1* mRNA levels correlate with prognostic indicators of aggressive breast cancer, lung metastases probability and triple negative breast cancer (TNBC). We establish that FMRP overexpression in murine breast primary tumours enhances lung metastasis while its reduction has the opposite effect regulating cell spreading and invasion. FMRP binds mRNAs involved in epithelial mesenchymal transition (EMT) and invasion including *E-cadherin* and *Vimentin* mRNAs, hallmarks of EMT and cancer progression.

## INTRODUCTION

One of the hallmarks of an aggressive tumour is its propensity to form metastases, and the understanding of this process is highly relevant to cancer treatment. The dissemination of cancer cells from primary tumours to form distant metastases is a highly regulated process consisting of invasion, intravasation, transit in the blood or lymph, extravasation and growth at a new site (Chaffer & Weinberg, 2011; Hanahan & Weinberg, 2011; Olson & Sahai, 2009; Sahai, 2007; Yilmaz & Christofori, 2009). The epithelial to mesenchymal transition (EMT) converts epithelial cells into migratory and invasive cells and is a fundamental event in both morphogenesis and cancer progression (Nieto, 2011; Nieto & Cano, 2012).

This transition is accompanied by increased cell motility, cytoskeleton remodelling and changes in cell adhesion properties, crucial events for tumour cell dissemination and metastasis formation as well as for neuronal development (Kim et al, 2009; Schmid & Maness, 2008). Those cellular events seem to be affected in patients with the fragile X syndrome (FXS), the most common form of inherited intellectual disabilities with an incidence of 1:2500 to 1:5000 in males and 1:4000 to 1:6000 in females (Bagni et al, 2012; Coffee et al, 2009; Turk, 2011). FXS is caused by the absence of the fragile X mental retardation protein (FMRP) and in neurons results in dendritic spine dysmorphogenesis possibly due to a dysregulated mRNA metabolism affecting cytoskeleton remodelling, synapses connections and shaping (Bagni et al, 2012; Bhakar et al, 2012; Gross et al, 2012).

Despite the role of FMRP has been very well established in brain, the protein is almost ubiquitously expressed, although at lower levels than in brain, suggesting that, in addition to its effects in the central nervous system, it may have fundamental roles in other organs and in other diseases. Previous works have underlined a link between FMRP and cancer. *FMR1* mRNA, encoding FMRP, is overexpressed in hepatocellular carcinoma cells (Li et al, 2003; Liu et al, 2007). Furthermore, a decreased risk of cancer has been reported in patients with FXS (Schultz-Pedersen et al, 2001), a decreased expression of the Wnt7A oncogene was detected in patients with FXS (Rosales-Reynoso et al, 2010) and a case study showed that a patient with FXS had an unusual decrease of tumour brain invasiveness (Kalkunte et al, 2007). However, a specific role for FMRP in regulating cancer biology, if any, remains unknown.

In this study we show, using a human tissue micro-array (TMA), that FMRP overexpression significantly correlates with prognostic indicators of aggressive breast cancer. Furthermore, high levels of *FMR1* mRNA in human breast tissues are associated with breast cancer metastatic to lungs and with triple negative breast cancer (TNBC).

Using a mouse model we establish that FMRP overexpression in breast primary tumours enhances lung metastasis while its reduction has the opposite effect regulating cell spreading from the primary tumour and invasion. Finally we show that in cancer cells FMRP binds mRNAs involved in EMT, cell adhesion and cytoskeleton remodelling and regulates their stability and translation.

## RESULTS

### FMRP is highly expressed in human breast cancer

**Figure 1:**
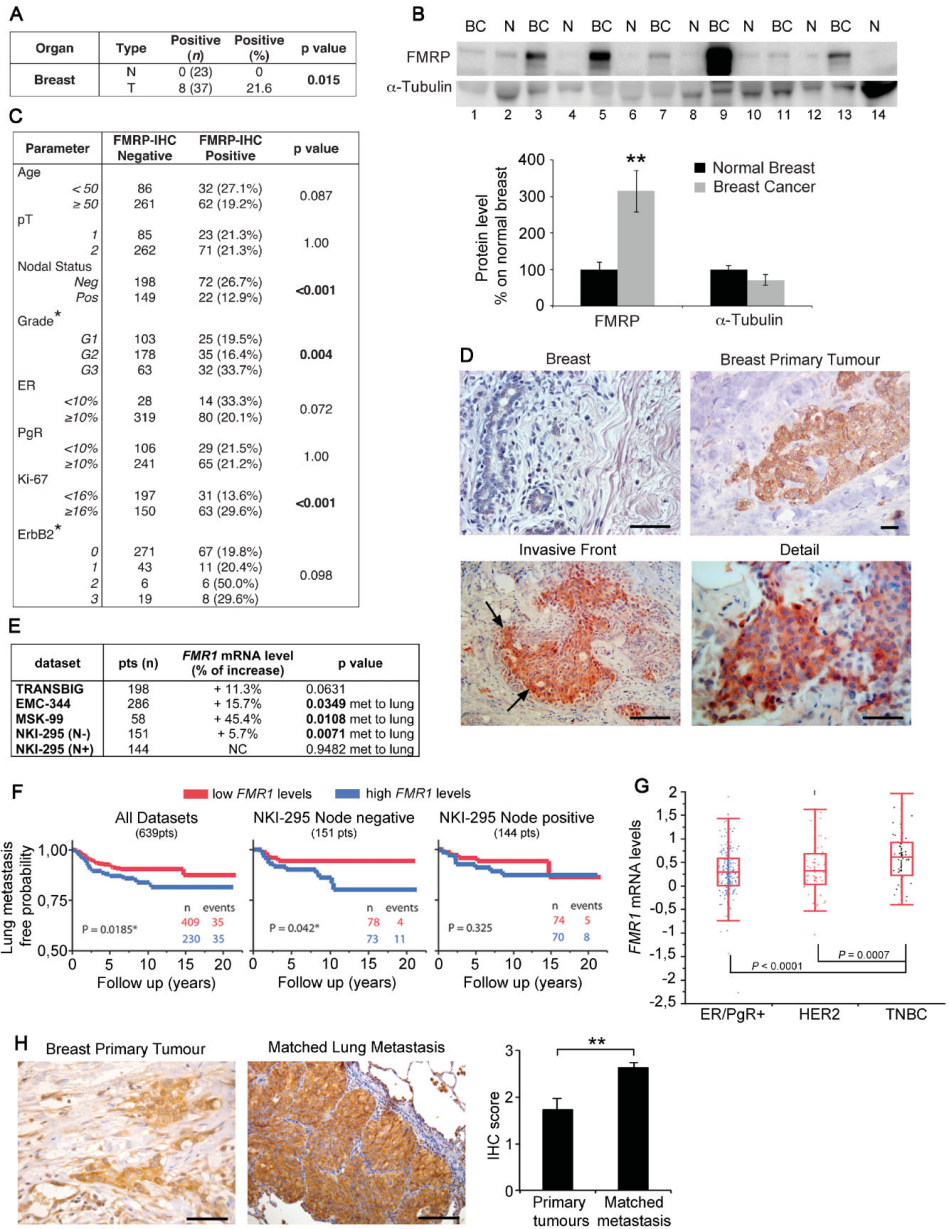
FMRP is highly expressed in human breast cancer and distal metastasis FMRP expression on human TMAs containing normal and multi-tumour tissues. (*n*), number of samples; (*N*), normal; (*T*), tumour tissue; (%), percentage of FMRP positive tissues (*p* = 0.015, contingency table analysis with Pearson chi-square test was applied (JMP™ IN 5.1).FMRP and α-tubulin expression in normal breast and ductal carcinoma revealed by Western blotting analysis. (*n* = 7 pairs, *p* < 0,01).FMRP expression on a TMA (Confalonieri et al, 2009) from human breast tumour (*n* = 477). *Not all clinical parameters were available. The number of cases with weak-moderate FMRP level (FMRP-IHC ≤ 1.0) and the number of cases with very high level of FMRP (FMRP-IHC > 1.0) as well as the percentage of FMRP positive cases (%) is reported in each patients subgroup and for each clinico-pathological parameter analysed. FMRP significantly correlates with high tumour grade (*p* = 0.004), high proliferation index (Ki67, *p* < 0.001) and negative lymph node status (*p* < 0.001).Representative images of FMRP expression in normal and tumour breast tissues including the margin of a breast primary tumour (overview and detail). Scale bars: 100 µm (panels 1-2-3) and 50 µm (panel 4).*FMR1* mRNA expression in breast primary tumours on four different breast cancer datasets: TRANSBIG (Desmedt et al, 2007), EMC-344 (Wang et al, 2005), MSK-99 (Minn et al, 2007) and NKI-295 (van de Vijver et al, 2002). Pts (*n*), number of patients. For the TRANSBIG dataset, *FMR1* mRNA level was calculated as a percentage of increased expression respect to non metastatic tumours, and for tumours nonmetastatic to lung for the EMC-344, MSK-99, NKI-295 cohorts using Welch's *t*-test. NC, not changed.Kaplan–Meier curves for the probability of having metastasis to lung. Pts, number of patients; *n*, number of patients in each subgroup: (low FMR1 mRNA levels in red and high levels in blue); events, number of patients with lung metastasis in those two subgroups. Left curve: all datasets metastatic to the lung (Pts = 639 excluding TRANSBIG), middle curve: NKI-295 node-negative dataset, right: NKI-295 node-positive dataset, *p*-values were calculated using the Log-rank test.*FMR1* mRNA expression was compared amongst the TNBC group (*i.e*. ER/PgR and HER2 negative) and the ER/PgR (*p* < 0.0001) and/or the HER2 positive (*p* = 0.0007) (Pts = 597).FMRP expression in primary breast tumours and matched lung metastases (*n* = 12, *p* < 0.01). Representative images of IHC for FMRP and quantification are shown. Scale bars: 50 μm.

An analysis of available expression datasets shows that *FMR1* mRNA is expressed in different tissues and in cancer cell types (https://www.genevestigator.com/gv/). To explore a possible role for FMRP in cancer biology, we examined FMRP expression level using a multi-tumour human TMA (Capra et al, 2006; Confalonieri et al, 2009) (Fig 1; Supporting Information Table S1A) with an FMRP specific antibody (Ferrari et al, 2007) (Supporting Information Fig S1). FMRP was significantly increased in breast tumours as compared to normal tissues that show a weak expression (Fig 1A). FMRP expression was also independently analysed on a panel of ductal carcinoma using the OncoPair INSTA-Blot™. FMRP resulted similarly increased in breast cancer tissues compared to normal breast, such a correlation was not observed for the protein α-tubulin (Fig 1B). Other tumour types showed similar findings (Supporting Information Table S1B). We further focused on breast cancer because it is the top cancer in women and, in some subtypes, has a poor prognosis (Coleman et al, 2008). FMRP expression analysis was carried out on a large collection (Supporting Information Table S2) of ductal and lobular breast cancer tissues (Confalonieri et al, 2009). Notably, FMRP was very highly expressed (scores > 1) in more than 20% of the breast primary tumour samples (Fig 1C; Supporting Information Fig S1) compared to normal tissue where it was expressed at lower levels. The histopathological evaluation showed the heterogeneity of FMRP expression in different tumour foci and at the margin (Fig 1D). The percentage of samples expressing high levels of FMRP correlates with high tumour grade (G3) and high proliferation index (Ki67) (Fig 1C), both of them indicators of poor prognosis (Elston & Ellis, 1991; Fitzgibbons et al, 2000; Goldhirsch et al, 2001). Finally, FMRP correlated with negative lymph node status.

On the basis of these findings we performed a gene expression analysis on four available breast cancer datasets that provide clinical information on the occurrence of distal metastasis. Analysis of the TRANSBIG cohort (Desmedt et al, 2007) revealed trend of increasing expression of *FMR1* mRNA in primary tumours that metastasize to distal organs (Fig 1E). In two other independent cohorts, *i.e*., EMC-344 (Wang et al, 2005) and MSK-99 (Minn et al, 2007), we found significantly increased *FMR1* mRNA expression in primary tumours that metastasize to lung (Fig 1E). *FMR1* expression correlates with lung metastases in the lymph node-negative subpopulation of the NKI-295 dataset (van de Vijver et al, 2002) while it does not in the lymph node-positive population (Fig 1E). Kaplan–Meyer curves generated by merging the three datasets for which the clinical information on pulmonary metastasis is available (EMC-344, MSK-99 and NKI-295) showed that high levels of *FMR1* mRNA correlated with an increased probability of metastasis to lungs (Fig 1F), but not to other distant organs (Supporting Information Fig S2). Cox proportional hazard analysis of the three cohorts revealed that patients with breast tumours overexpressing *FMR1* mRNA have an increased risk to develop lung metastasis (hazard ratio (HR) = 1.21; 95% CI 1.02–1.45, *p* = 0.0293) and this is independent from estrogen receptor status (HR = 1.51 95% CI 1.27–1.85, *p* < 0.0001), the only pathological parameter available for all datasets considered. This suggests that FMRP increased expression might have a role in metastatic spreading of breast tumour cells to the lungs. We next analysed *FMR1* mRNA expression on a large cohort of breast cancer patients (Cancer Genome Atlas Network, 2012) recently made available by the Tumor Cancer Genome Atlas consortium (TCGA). Strikingly, *FMR1* mRNA expression was increased with a high statistical significance in the more aggressive TNBC subtype (*i.e*. ER/PgR and HER2 negative) compared to the ER/PgR and/or the HER2 positive tumours (Fig 1G). Due to lack of information regarding distant metastasis in this cohort we could not perform further studies. TNBCs, although clinically more aggressive, are more likely to metastasize at distant site such as lung (Brouckaert et al, 2009; Van Belle et al, 2009) independently of having involved lymph nodes at diagnosis (Hudis & Gianni, 2011; Reddy, 2011).

Finally, an increased expression of FMRP in lung metastases was independently verified on paired cases of human breast primary tumours and matched lung metastases (Fig 1H and Supporting Information Table S3).

Overall, these findings suggest an association of FMRP overexpression to breast cancer progression, and in particular to the metastatic spread to the lungs.

Finally we assessed the occurrence of breast cancer in a cohort of women from the FXS population in England for which cancer clinical history is also available, an informative cohort for this study since it is quite rare to have access to both data at the same time (Supporting Information Table S4). Five patients with different cancer types were identified, significantly less than the expected 15.93 given the national cancer incidence rate in England. Only one case of breast cancer was present compared to an expected of 5.79. However, due to the lack of information on distal events, we could not monitor cancer progression in those patients with FXS. Notably, these findings further extend previous studies of a reduced incidence of cancer in a Danish cohort of FXS patients (*n* = 223) (Schultz-Pedersen et al, 2001).

### FMRP levels affect the formation of lung metastasis

**Figure 2:**
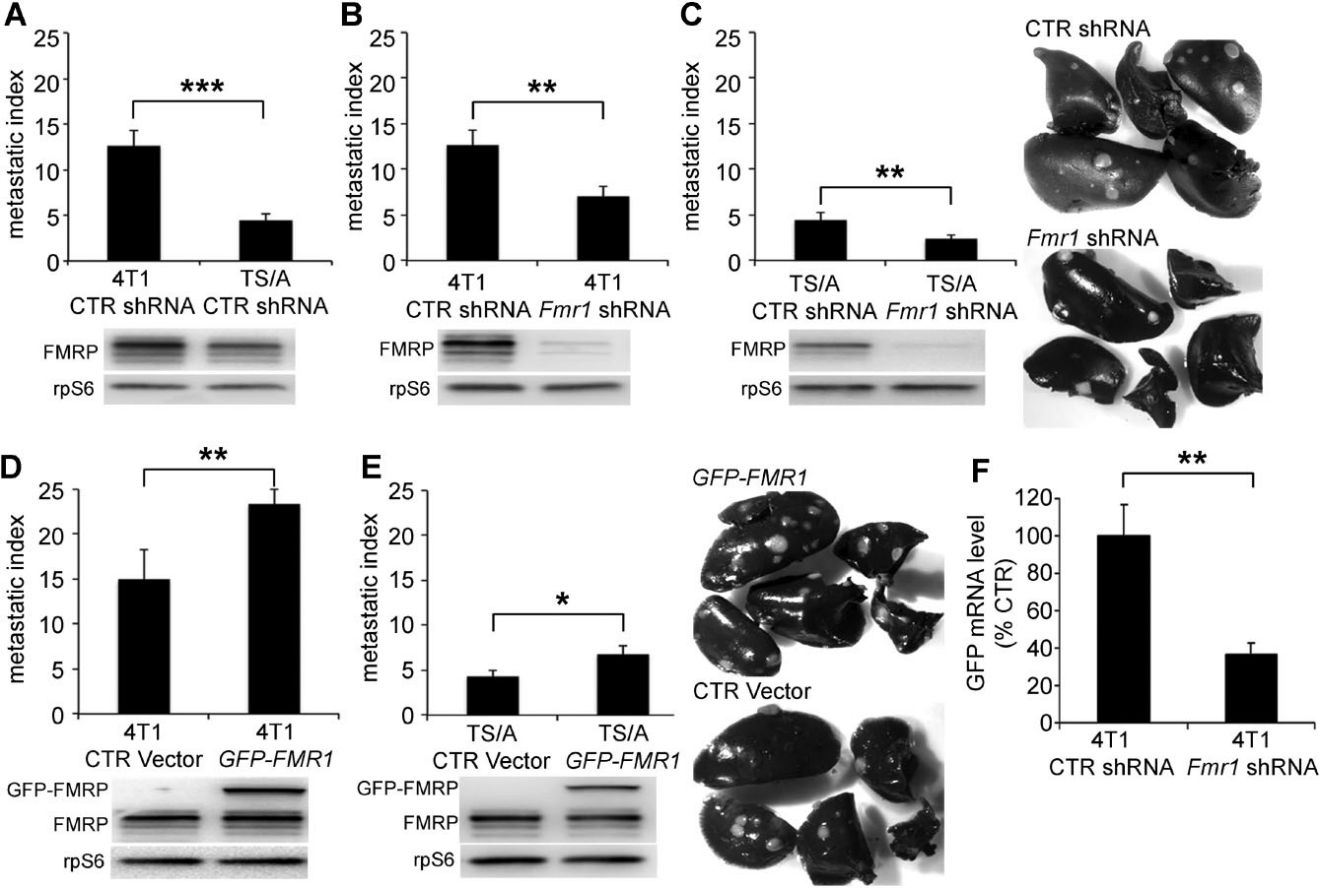
Fmrp levels influence metastasis formation Metastatic index (number of lung metastases per tumour weight) after orthotopic injection (O.I.) of control (CTR shRNA) 4T1 and TS/A cells (*n* = 13 and 12, respectively, *p* < 0.001). Fmrp expression in control cells (CTR shRNA) is shown relative to the ribosomal protein S6 (rpS6), representative Western Blotting (WB).Metastatic index after O.I. of CTR shRNA and *Fmr1* shRNA (3/4) 4T1 cells (*n* = 13, *p* < 0.01). Representative Western blotting (WB) for Fmrp and rpS6 is shown.Left as in panel (B) using TS/A cells (*n* = 12, *p* < 0.01). Representative WB and lungs with metastases (white spots).As in (B) using 4T1 cells expressing the control vector or overexpressing FMRP (*GFP-FMR1*). Analysis was performed after O.I. (*n* = 6, *p* < 0.01). Representative WB is shown.As in (D) using TS/A cells (*n* = 12, *p* < 0.05), shown WB for Fmrp and representative lungs.Number of circulating cancer cells expressed as ratio of GFP-*Fmr1* shRNA *versus* GFP-CTR shRNA cells (*n* = 10, *p* < 0.01).

To verify that Fmrp levels affect tumour progression, we used two murine breast cancer cell lines with different metastatic properties, 4T1 (Tao et al, 2008) and TS/A (Nanni et al, 1983), and with different levels of Fmrp expression (Fig 2A and Supporting Information Fig S3). CTR shRNA and *Fmr1* shRNA cells were orthotopically injected into the mammary fat pad of syngenic mice. The generated tumours showed comparable growth, with a small difference at the end of the time course (Supporting Information Fig S3E and F). Tumours also showed higher levels of Fmrp and *Fmr1* mRNA compared to healthy breast tissues (Supporting Information Fig S4A–C). Importantly, tumours derived from 4T1 CTR shRNA cells formed a significantly higher number of lung metastases compared to TS/A CTR shRNA cells (Fig 2A and Supporting Information Fig S4D). Reduction of Fmrp expression decreased the metastatic index formed by both cell lines by 50%, relative to their respective control cells (Fig 2B and C) while FMRP overexpression resulted in an increase by 56% in the 4T1 (high metastatic potential) and by 72% in the less metastatic cell line TS/A (low metastatic potential) (Fig 2D and E).

To establish the effect of Fmrp on tumour kinetics we used the 4T1 cell line expressing GFP and silenced for Fmrp (GFP-*Fmr1* shRNA, Supporting Information Fig S5A). Mice orthotopically injected with *Fmr1* silenced cells have less circulating cancer cells compared to control (Fig 2F) as detected by *GFP* mRNA levels (Schuster et al, 2004). We next monitored cell survival in the bloodstream and cell lodging in the lungs after tail vein injection. As shown in Supporting Information Fig S5B and C, no difference between the two cell lines was observed.

### FMRP levels affect cell–cell adhesion, cell shape and invasion of 4T1 cell lines

**Figure 3:**
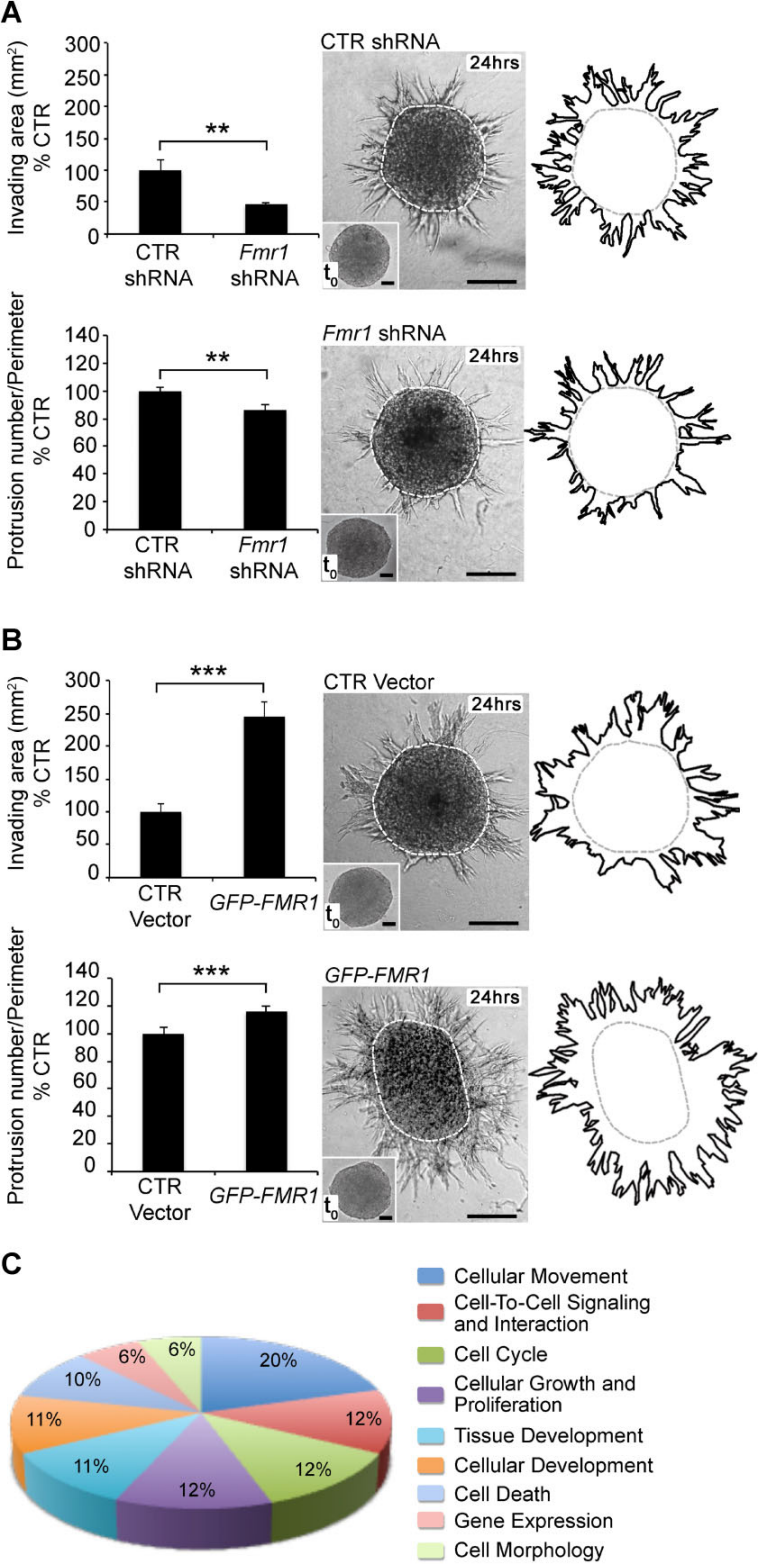
Fmrp levels affect cell invasion 3D multicellular tumour spheroids invasion assay. Phase-contrast images show the 3D spheroids from CTR shRNA and *Fmr1* shRNA (3/4) 4T1 cells, white dotted line indicates the spheroid body. Histograms represent the quantification of the invading area and the number of protrusion (*n* = 20 per condition, *p* < 0.01). Scale bar = 200 μm. All insets show time = 0. Scale bar: 50 μm.Same as in (A) with CTR vector and GFP-*FMR1* 4T1 cells (*n* = 20 per condition, *p* < 0.001).Ingenuity pathway analysis of the Fmrp target mRNAs (*p* < 0.05).

We next investigated the cell–cell adhesion property of tumour cells with different Fmrp levels upon Ca^2+^ deprivation (Kim et al, 2011; Silva et al, 2009; Wilby et al, 1999). Fmrp-depleted cells keep their cell–cell adhesion while FMRP overexpressing cells (*GFP-FMR1*) detach from neighbouring cells and change shape (Supporting Information Fig S6A). Furthermore, cells expressing Fmrp have different cell area compared to *Fmr1* shRNA cells (Supporting Information Fig S6B) and an increased propensity to migrate through a monolayer of endothelial cells (Supporting Information Fig S6C). Finally when the 4T1 cells were cultured as 3D cell spheroids (Del Duca et al, 2004; Hattermann et al, 2011) those overexpressing Fmrp exhibited more protrusions and increased cell area when compared to Fmrp-silenced cells (Fig 3A–B and Supporting Information Fig S6D for different *Fmr1* shRNA combinations). Of note, changes in cell shape and migration properties are hallmarks of EMT (Thiery et al, 2009).

### EMT related molecules are present in the Fmrp complex

While in brain FMRP regulates a subset of neuronal mRNAs, FMRP-associated mRNAs in cancer, if any, have not been identified yet. We immunoprecipitated the Fmrp complex using specific FMRP antibodies (Ferrari et al, 2007) and Supporting Information Fig S1 from 4T1 cells (Supporting Information Fig S7) and tumour tissues (unpublished observations) and analysed the EMT RT^2^ Profiler™ PCR Array (see materials and methods section). 42 mRNAs, out of 84 analysed specifically co-precipitate with Fmrp. The Fmrp interacting mRNAs were then grouped using IPA (Ingenuity® Systems) that, through an interactive analysis of complex “omics” data, allowed us to investigate, with a statistical significance, all available cellular pathways and functions (Fig 3C and Supporting Information Table S5). Most of the FMRP targets encode for proteins involved in cellular movement, migration and motility, adhesion and EMT such as Vimentin (*Vim*), E-cadherin (*Cdh1*), Microtubule associated protein 1B (*Mtap1b*), Occludin (*Ocln*), or involved in cancer signal transduction such as estrogen receptor 1 alpha (*Esr1*), epidermal growth factor receptor (*Egfr*), notch gene homologue 1 (*Notch 1*) or transcription factors such as twist homologue 1 (*Twist1*), fibronectin 1 (*Fn 1*) and zinc finger E-box binding homoeobox2 (*Zeb2*). Very similar results were obtained by immunoprecipitating FMRP from tumours generated by orthotopic injection (unpublished observations). E-cadherin, a cell–cell adhesion molecule, and Vimentin, a major constituent of the intermediate filament family of proteins, were particularly interesting because the two proteins play a key role in EMT. During this process epithelial markers like E-cadherin are downregulated, while mesenchymal markers like Vimentin are increased with consequent acquisition of an invasive capacity (Kalluri & Weinberg, 2009; Lahat et al, 2010).

### E-cadherin and Vimentin expression is regulated by FMRP

**Figure 4:**
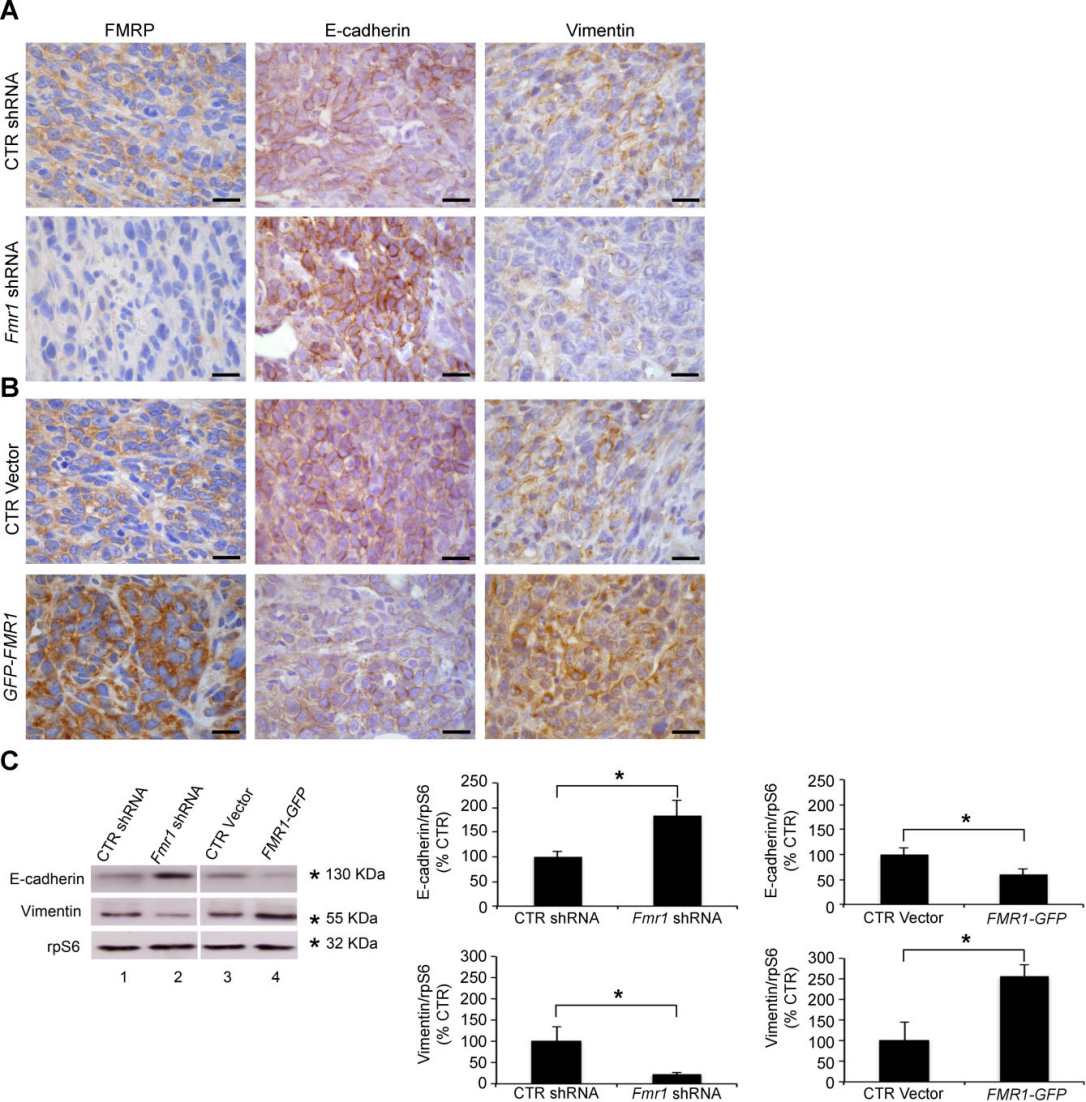
In breast tumours E-cadherin and Vimentin are regulated by Fmrp Representative IHC for Fmrp, E-cadherin and Vimentin on tumours generated by control (upper panels) and *Fmr1* shRNA 4T1 cells (lower panels). Scale bars 200 μm.Same as in (A) with cells expressing CTR vector and overexpressing *FMR1 mRNA*.Quantification of the Western blot analysis for Vimentin and E-cadherin from tumours generated by O.I. of CTRs, *Fmr1* shRNA and FMRP overexpressing 4T1 cells (*n* = 5, *p* < 0.05, Student's *t*-test). A representative Western blot is shown.

**Figure 5:**
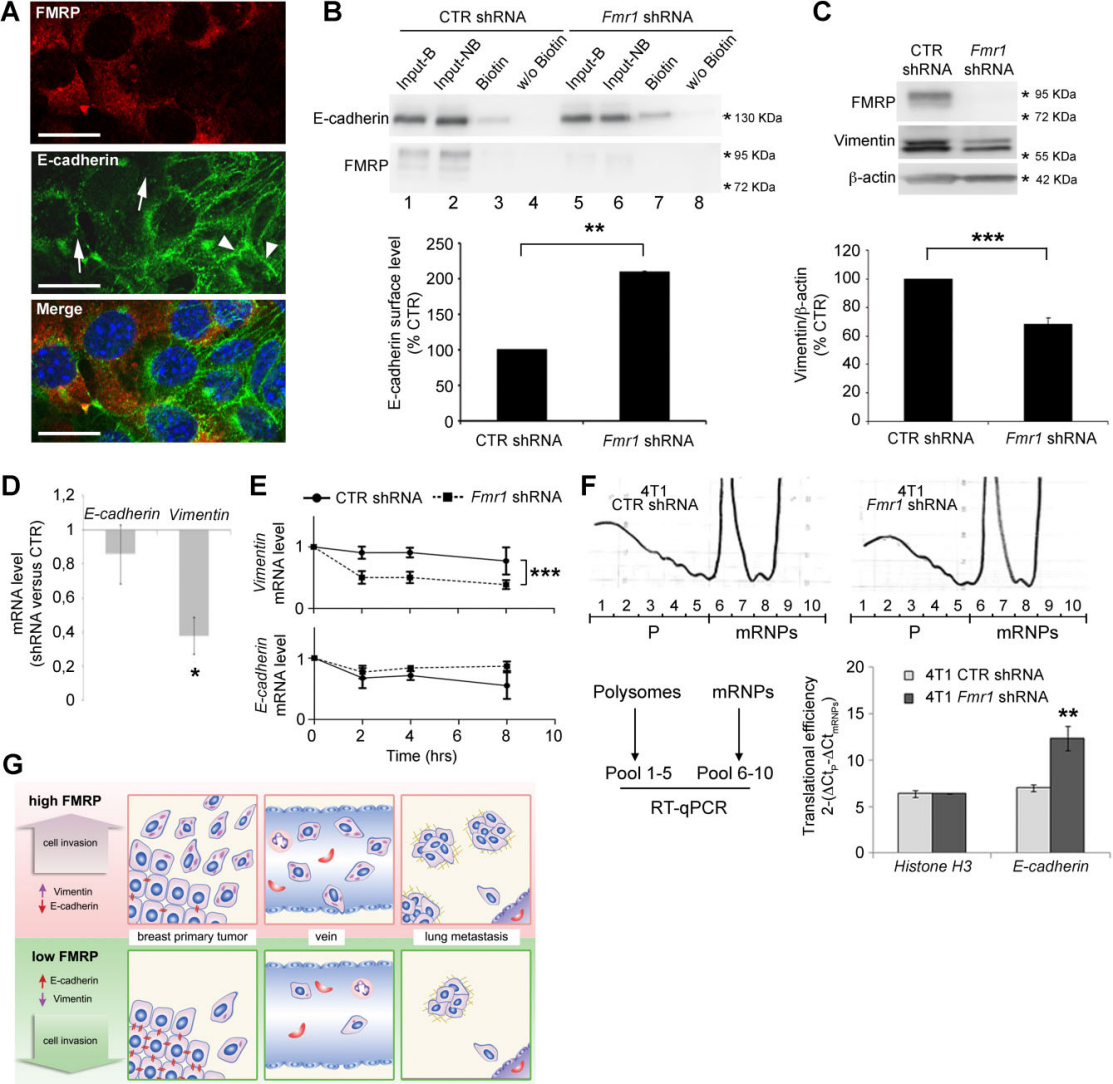
Fmrp regulates E-cadherin and Vimentin in breast cancer cells Fmrp (red) and E-cadherin (green) detection by I.F. in 4T1 cells expressing different Fmrp levels. Nuclei are visualized by DAPI staining. Arrows point to cell-cell junctions in 4T1 cells expressing Fmrp (low E-cadherin), while arrowheads point to junctions of cells with reduced Fmrp level (high E-cadherin). Scale bar = 10μm.Western blotting of surface proteins from CTR shRNA and *Fmr1* shRNA cells. Lanes 1 and 5 show the total protein extract (1/50) of biotin treated cells, lanes 2 and 6 same from non-treated cells. The respective streptavidin precipitates are shown in lanes 3, 4, 7 and 8. E-cadherin level in 4T1 shRNA and CTR shRNA cells (*n* = 3, *p* < 0.01, Student's *t*-test).Vimentin protein level in CTR shRNA and 4T1 shRNA 4T1 cells revealed by Western blotting (*n* = 4, *p* < 0.001, Student's *t*-test).*E-cadherin* and *Vimentin* mRNA levels in 4T1 CTR shRNA and *Fmr1* shRNA cells detected by RT-qPCR (*n* = 3, *p* < 0.05).mRNA stability assay in 4T1 CTR shRNA and *Fmr1* shRNA cells. RNA was isolated at the indicated time points after Actinomycin D treatment and the stability of *Vimentin* (upper panel) or *E-cadherin* (lower panel) mRNAs was analysed by RT-qPCR (*n* = 7, *p* < 0.001).Translational efficiency analysis. Upper panel, polysome-mRNPs distribution on a sucrose gradient. Low left panel, fractions 1–5 corresponding to translating polysomes (P) and fractions 6–10 corresponding to silent mRNPs were pooled. Low right panel, quantification of the translational efficiency of *Histone H3.3* and *E-cadherin* mRNAs reported as ratio of P over mRNPs (2^−[ΔCt(P)–ΔCt(mRNPs)]^) (*n* = 4, *p* < 0.01).Working model. FMRP levels regulate the expression of mRNAs involved in cell invasion. Upper row, high levels of FMRP lead to increased metastases formation. Lower row, opposite effect is observed in absence of FMRP.

Immunohistochemistry revealed that expression of E-cadherin and FMRP were inversely correlated, while FMRP and Vimentin levels were directly correlated in both human and mouse tumour tissues (Fig 4A and B and Supporting Information Fig S8). Western blotting analysis on the generated tumours confirmed these findings (Fig 4C). Furthermore, 4T1 cells with reduced Fmrp levels have an increase of functional E-cadherin on the cell surface (Fig 5A and B) and a decreased Vimentin (Fig 5C).

In brain FMRP has been widely studied for its function as regulator of mRNA metabolism. In particular FMRP can act as negative regulator of translation (Bassell & Warren, 2008; Darnell et al, 2011; Napoli et al, 2008) or can stabilize messenger RNA (D'Hulst et al, 2006; De Rubeis & Bagni, 2010; Miyashiro et al, 2003; Zalfa et al, 2007), depending on the identity of the target mRNA and the cellular context.

FMRP target mRNAs in cancer cells have not been identified yet and consequently the molecular mechanism/s through which FMRP regulates its specific mRNA targets. Since *E-cadherin* and *Vimentin* are bona fide cell invasion and metastasis mRNAs (Cano et al, 2000; Cowin et al, 2005; Huber et al, 2011; Kallergi et al, 2011; Korsching et al, 2005; Lahat et al, 2010; Rakha et al, 2005; Thiery et al, 2009; Willipinski-Stapelfeldt et al, 2005; Yoo et al, 2012) involved in the initial steps of cancer progression we further investigated their Fmrp-mediated regulation.

The level of *E-cadherin* mRNA was analysed by RT-qPCR using CTR and *Fmr1* silenced 4T1 cells and did not reveal any change in the mRNA steady state (Fig 5D) while a decrease in *Vimentin* mRNA was observed (Fig 5D), consistent with the decreased Vimentin protein level (Fig 5C). To address if the mechanism leading to changes in *Vimentin* mRNA levels could be due to a regulation of transcription or mRNA stability, we treated CTR and *Fmr1* shRNA 4T1 cells with the transcriptional suppressor Actinomycin D. Upon 2 h treatment, cells depleted of FMRP showed a reduced *Vimentin* mRNA levels, that remained down regulated (see time points), proving that FMRP is indeed regulating its stability. A similar regulation was observed for other mRNAs associated to FMRP (Supporting Information Table S5) and key EMT markers such as *Fibronectin 1 (Fn1), Jagged 1 (Jag1), Matrix Metallopeptidase 9 (MMP9), Serine (or Cysteine) Peptidase Inhibitor, Clade E, Member 1 (Serpine 1), Epidermal Growth Factor Receptor (Egfr)* (Supporting Information Fig S9). *E-cadherin* mRNA, whose level does not change in absence of Fmrp, did not show any response to the treatment (Fig 5E) suggesting a possible control at the level of its translation.

In brain FMRP is largely involved in mRNA translational regulation (Bagni & Greenough, 2005; Bagni et al, 2012; Bassell & Warren, 2008), we then analysed the polysome-mRNP distribution (Napoli et al, 2008; Zalfa et al, 2003) (translational efficiency) of *E-cadherin* mRNA in CTR and *Fmr1* shRNA cells. As shown in Fig 5F, *E-cadherin* mRNA was translated at a higher efficiency in the absence of Fmrp in agreement with its role as translational repressor. Additional mRNAs, involved in EMT and target of FMRP in cancer cells (Supporting Information Table S5), such as *Microtubule Associated Protein 1B (Mtap1b), Caveolin 2 (Cav2), Desmoplakin (Dsp), Keratin 14 (Krt14), Microphthalmia-Associated Transcription Factor (Mitf)* were similarly regulated at the level of translation (Supporting Information Fig S10). The effects that we observe at the level of mRNA metabolism changing the level of Fmrp were not due to an off target effect since they were reproduced silencing *Fmr1* with different siRNAs (Supporting Information Fig S11A and B).

## DISCUSSION

Acquisition of the correct metastatic signature, the precise nature of which is mostly unknown, confers an advantage for cancer cells to survive and metastasize. The ability of tumour cells to form metastases requires adaptive changes in their shape and adhesive repertoire and acquisition of motility that is critical for both escape from the primary tumour and colonization (Hanahan & Weinberg, 2011; Yilmaz & Christofori, 2009).

Metastatic progression can be enhanced not only by above-mentioned qualitative changes but also quantitative alterations of metastasis-associated mRNAs (Graff & Zimmer, 2003; Kang & Massague, 2004). It is well established that mRNA metabolism and translational control largely contribute to cancer progression (Hsieh et al, 2012; Silvera et al, 2010; Stumpf & Ruggero, 2011).

In brain FMRP modulates the expression of selected mRNAs (Bagni & Greenough, 2005; Bassell & Warren, 2008; Darnell et al, 2011; De Rubeis & Bagni, 2010) in two ways: FMRP can enhance mRNA stability of certain mRNAs and can also block their translation (D'Hulst et al, 2006; Zalfa et al, 2007).

The dataset we generated upon specific immunoprecipitation of Fmrp (Fig 3 and Supporting Information Table S5) suggests that FMRP acts as a master regulator of a large subset of mRNAs involved in multiple steps of cancer progression including invasion and intravasation: two of the several steps in tumour progression. Recently multiphoton imaging of tumours *in vivo* revealed that entry into circulation is a critical step of metastasis (Wyckoff et al, 2011). Here we studied in detail the gene regulation mediated by Fmrp on several of the identified mRNA targets such as *Vimentin, Fibronectin 1, Jagged 1, Matrix Metallopeptidase 9, Serpine 1, Epidermal Growth Factor Receptor, E-cadherin, Microtubule Associated Protein 1B, Caveolin 2, Desmoplakin, Keratin 14, Microphthalmia-Associated Transcription Factor*. We show that some of these mRNAs are regulated at the level of stability, while others are translationally repressed in agreement with the previous described dual function of FMRP in neurons (Bagni et al, 2012). Recent studies have demonstrated that translation deregulation contributes to the metastatic phenotype through selective effects on the translation of mRNAs whose products are involved in various steps of the metastatic process (Nasr & Pelletier, 2012). With the present study we suggest that FMRP might be indeed a key player in mRNA metabolism and tumour progression.

Amongst its target, FMRP controls E-cadherin and Vimentin levels, important molecules for cell adhesive properties, cytoskeleton remodelling and consequently tumour cell behaviour.

Both reduced E-cadherin and overexpression of Vimentin are observed during EMT and cancer progression (Berx & van Roy, 2009; Kang & Massague, 2004), leading to the shedding of the cancerous cells from the primary tumour (Hanahan & Weinberg, 2011; Satelli & Li, 2011; Schmalhofer et al, 2009; Thiery et al, 2009; Yilmaz & Christofori, 2009). Although *Vimentin* has still an elusive function in invasive migration is routinely used as mesenchymal marker during the transition.

We propose that Fmrp repression of *E-cadherin* (high Fmrp level) and other mRNA targets encoding for proteins that prevent tumour shedding together with an increase in proteins promoting invasion would support the enhanced ability of FMRP positive cells to detach from the primary tumour and invade allowing tumour spreading and subsequent metastases formation (Fig 5G). Therefore, in the absence of FMRP (FXS) the increase of the E-cadherin and decrease in Vimentin, exemplary of the FMRP mediated regulation, would result in the protective metastatic phenotype.

In human, this model is further supported by the correlation of FMRP with prognostic indicators of cancer dissemination (Fig 1) and with the reduced risk of cancer incidence in patients with FXS (Supporting Information Table S4). Moreover, FMRP expression is higher in TNBC compared to ER/PgR and/or Her2 positive tumours. TNBC, that are more likely to form metastases at distant sites like lungs (Brouckaert et al, 2009) independently of having lymph nodes involved at diagnosis (Hudis & Gianni, 2011; Reddy, 2011), have a poor prognosis and characteristics of EMT (Jeong et al, 2012).

Changes in cell-to-cell signalling and interaction mediate the switch between epithelial and mesenchymal phenotypes and consequently dictate the receptivity towards signals from the extracellular milieu. These signals include soluble growth factors, receptors, cytokines and extracellular matrix. Notably, FMRP target mRNAs identified in this study encode for several of these molecules.

From this work we provide evidence that FMRP regulates the same classes of genes in brain and breast cancer, which indicates that the protein has not acquired a novel function in tumours. FMRP targets account for 4% or 27% of the transcriptome, according to the cell type (Ascano et al, 2012; Brown et al, 2001; Darnell et al, 2011). Among those mRNAs, there are several encoding for factors involved in cell shape, cell-adhesion and invasion properties including Vimentin (Miyashiro et al, 2003), Microtubule associated protein 1B (Brown et al, 2001; Miyashiro et al, 2003; Zalfa et al, 2003), Transforming Growth Factor 2 (Miyashiro et al, 2003), LI-cadherin (Miyashiro et al, 2003), Neuronal Cell Adhesion Molecule (Darnell et al, 2011) and Catenin beta1 (Darnell et al, 2011), Matrix Metallopeptidase 9 (Bilousova et al, 2009). Of note, more than 50% of the FMRP target identified in this study were independently found to be directly associated to FMRP in HEK293 cells (Ascano et al, 2012) while 10% are in common with another study in hippocampus (Darnell et al, 2011). Importantly, EMT is known to play a role in neuronal crest migration and it is therefore tempting to suggest that some of the pathology observed in patients with FXS may be related to the role we uncovered that FMRP plays in EMT.

## MATERIALS AND METHODS

### Human tissues collection

Studies described in this paper and involving humans and animal models have been performed upon approval of European Committees and with informed consent from the patients. All experiments involving human specimens conformed to the principles described in the NMA declaration of Helsinki and the NIH Belmont report. Patients' recruitment and tissue collections are described in Supporting Information.

### Immunohistochemical analysis of FMRP on human tissue microarrays (TMA)

Samples were provided by the Pathology Departments of Ospedale Maggiore (Novara), Presidio Ospedaliero (Vimercate), Ospedale San Paolo (Milan) and Ospedale Sacco (Milano) and analysed on multi-tumour TMAs (see Supporting Information).

### Microarray and statistical analyses

Affymetrix Microarray data and relative clinical and pathological information were downloaded from GEO (Gene expression Omnibus, http://www.ncbi.nlm.nih.gov/geo/) using the accession number GSE7390 for the TRANSBIG dataset, GSE2034 for the ERASMUS dataset, GSE5327 for the MSK-99 dataset, and NKI-295 at http://www.rii.com/publications/2002/nejm.html. Data were generated using the MAS5.0 and processed in GeneSpring 7.3 (Agilent).

Statistical analyses were performed on log2 median centred data using JMP IN 5.1 (SAS) and Welch's *t*-test. See Supporting Information for Kaplan Mayer curves.

Gene expression data matrix (Level 3 data) of breast invasive carcinoma screening performed by the TCGA consortium was downloaded from (https://tcga-data.nci.nih.gov). Dataset consists in 597 breast cancer patients with annotated clinical and pathological information. Student's t-test analysis was performed.

FMRP expression in the TMAs was performed using Contingency Table analysis with Pearson chi-square test (JMP™ IN 5.1). In the breast cancer TMA (*n* = 477 patients) the association between the clinical-pathological variables of the tumours and FMRP expression was evaluated by Fisher's exact test or Likelihood Ratio test when more than two parameters were considered.

Student's *t*-test was used to study the expression of FMRP in IHC samples (tumours and matched distal metastasis).

Cancer incidence in the FXS population was calculated using Poisson distribution and one-sided test.

For the experiments performed in Figs 4 Student's *t*-test was applied except for the Ingenuity Pathway Analysis (Fig 3C, *p* < 0.05, Fisher's Exact test) and stability assay (Fig 5E, Two-Way ANOVA with Bonferroni correction). A *p*-value of less than 0.05 was considered as significant.

**The paper explained**

PROBLEM:

Breast cancer is the most common cancer in women worldwide with a lifetime risk of 1 in 8. The cause of patients' death with TNBC is often recurrence that occurs in 30–40% of the cases within 5 years from the surgery. Chemotherapy remains the only possible option and for this reason the identification of molecular events underlying TNBCs and breast cancer metastasis is needed to develop an efficient therapy.

Individuals with intellectual disabilities show a difference in cancer incidence according to the cancer type, the majority tend to have a significantly increased risk of cancer, patients with the Fragile X Syndrome, on the contrary, show a decreased cancer incidence.

RESULTS:

We show that the Fragile X Mental Retardation Protein is upregulated in highly metastatic human breast tumours. FMRP as well as FMR1 mRNA levels correlate with prognostic indicators of aggressive breast cancer and lung metastasis. Furthermore, reduction of FMRP in murine tumour cells decreases their ability to form lung metastases as a result of decreased cell invasion, while its overexpression increases metastatic potential. Finally, we identified specific FMRP target mRNAs involved in epithelial to mesenchymal transition, often a prerequisite for metastases formation, and show that FMRP controls their mRNA metabolism.

IMPACT:

Despite marked advances in breast cancer screening and treatment over the past 30 years, there is a need to develop new therapies in particular for cancer cases marked by distant events. Understanding the molecular mechanisms regulating metastasis dissemination might contribute to advance the treatment of very aggressive breast cancer such as TNBCs. Our findings highlight a novel function for the Fragile X Mental Retardation Protein in regulating mRNA metabolism of cancer genes and laid the first stone for further research on the molecular events of metastatic dissemination FMRP-mediated.

### IHC analysis on mouse tissue

Murine tissues were stained on a Bond-maxTM fully automated staining system (Leica Microsystems GmbH, Germany, see Supporting Information).

### Tumour cell lines

4T1 and TS/A cells (CTRs, *Fmr1* shRNA and overexpressing Fmrp) were grown in DMEM media containing fetal bovine serum 10% and 1% penicillin–streptomycin (Invitrogen) and kept at 37°C in 5% CO_2_.

### Orthotopic injection (O.I.) in mice

Lentivirus infected 4T1 and TS/A cells (combination of two independent *Fmr1* shRNAs 3 and 4; control (CTR) scrambled shRNA; *GFP-FMR1*; control (CTR) vector were used (see Supporting Information).

### Intravasation assay

1 × 10^6^ GFP labelled 4T1 cells were injected in the right second thoracic mammary fat pad of 9 weeks old Balb/c female mice. 29 days after injection 400 μl of blood were collected by retro-orbital bleeding and subjected to haemolysis. RNA was extracted using QIAamp RNA Blood Mini Kit (QIAGEN) following the manufacturer's instruction (see Supporting Information).

### 3D multicellular tumour spheroid invasion assay

CTR shRNA, *Fmr1* shRNA (3/4, 4/5 and 3/5) and *FMR1* overexpressing 4T1 cells were grown as pending drops, embedded in a collagen type I matrix and imaged after 24 h (see Supporting Information).

### RNA extraction, immunoprecipitation, RT-qPCR array, and Western blotting

These methodologies are described in Supporting Information.

### RNA stability assay

4T1 CTR and *Fmr1* shRNA cells were treated at *t* = 0 with Actinomycin D (1 µg/ml) for 0, 2, 4, 8 h. RNA was extracted and RT-qPCR performed as described in Supporting Information.

### Polysome-mRNPs distribution

Polysome-mRNPs distribution on a sucrose gradient was performed according to (Napoli et al, 2008). Cytoplasmic extracts from CTR and *Fmr1* shRNA 4T1 cells were fractioned by centrifugation on a 15–50% sucrose gradient. 10 fractions were collected while 254 nm absorbance was recorded (see Supporting Information).

## For more information

Associazione Italiana Sindrome X Fragile (http://www.xfragile.net)

Conquer Fragile X (http://www.conquerfragilex.org/)

FRAXA Research Foundation (http://fraxa.org/)

National Fragile X Foundation (http://www.fragilex.org)

## Supplementary material

As a service to our authors and readers, this journal provides supporting information supplied by the authors. Such materials are peer reviewed and may be re-organized for online delivery, but are not copy-edited or typeset. Technical support issues arising from supporting information (other than missing files) should be addressed to the authors.

Review Process File

**Figure S1.** (A) Two consecutive sections of a breast tumour TMA were stained with the same concentration of specific FMRP affinity purified antibodies (Ferrari et al, 2007) or the corresponding affinity-purified pre-immune IgGs. Shown are representative images. The breast tumour core was scored positive for FMRP using the specific antibody and negative with the pre-immune purified IgG. (B) Western blot analysis of FMRP expression in WT and *Fmr1* KO mouse brain using specific Fmrp/FMRP antibodies (Ferrari et al, 2007). The signal appears clear in WT extracts and absent in *Fmr1* KO extracts, showing specificity. (C) Immunohistochemistry detection of FMRP in formalin-fixed and paraffin-embedded mouse brains using in parallel the specific FMRP antibodies (affinity-purified IgGs) and the affinity purified IgGs from the preimmune serum. The regions in the hippocampus known to contain FMRP are strongly highlighted showing the specificity of the anti-FMRP antibodies in immunohistochemistry. Scale bars: 500 μM (left panels) and 100 μM (right panels). (D) FMRP scoring on normal and tumour breast tissues on TMAs. Representative images of IHC for FMRP performed on different human breast tissues that show the range of FMRP staining (low ≤1 to very high = >1). Scale bars = 100 μm.

**Figure S2.** Kaplan–Meier curves of metastasis free probability. Left curve, EMC-344, MSK-99, NKI-295 datasets correlating *FMR1* mRNA levels with the probability of having metastasis to distal organs (lung excluded). *FMR1* low levels are indicated in red while high levels in blue. Middle curve: same as the left curve using only the NKI-295 node-negative dataset. Right curve: same as left curve using only NKI-295 node positive dataset. Pts = total number of patients analysed (*n* = 569). *p*-values were calculated using the Log-rank test. *n* = number of patients; events = number of patients with distal metastasis. High levels of *FMR1 mRNA* do not correlate with an increased probability of lung metastasis to distant organs.

**Figure S3.** (A) FMRP expression in mouse breast tumour cell lines (4T1, TS/A, *n* = 5, *p* < 0.01). FMRP levels were analysed by Western blot using specific FMRP antibodies (Ferrari et al, 2007) and normalized for Vinculin and Comassie staining (not shown). Quantification is reported in the histogram as ratio FMRP/Vinculin where FMRP levels in 4T1 cells were considered 100%. (B) Silencing of *Fmr1* in 4T1 cells. 4T1 cells were transiently transfected with five different shRNAs as well as with a scrambled shRNA (see Methods). Fmrp levels were detected by Western blot, normalized to Vinculin and values reported in the histogram as ratio Fmrp/Vinculin where Fmrp levels in CTR cells were considered 100%. (C) 4T1 cells silenced for FMRP. Cells were stably transduced with different combination of three shRNAs against the *Fmr1* gene. Fmrp levels were detected by Western blot and normalized to Vinculin or total proteins (Coomassie staining, data not shown). Fmrp levels are expressed as a ratio to control cells (100%). (D) Same as panel (C) for the TS/A cell line. (E) Primary tumour growth after orthotopic injection of control (CTR) and *Fmr1* silenced 4T1 cells in WT syngenic mice (*n* = 12, *p* = 0.09, two-way ANOVA applying the Bonferroni correction). The graph represents tumour volume as a function of time after the injection. (F) Primary tumour growth after orthotopic injection of control (CTR) *and Fmr1* silenced TS/A cells in WT syngenic mice (*n* = 13, *p* = 0.23, two-way ANOVA applying Bonferroni correction). The graph represents tumour volume as a function of time after the injection.

**Figure S4.** (A) IHC analysis of Fmrp expression in primary tumours generated by orthotpic injection of CTR or *Fmr1* shRNA TS/A cells. Shown is an overview (left and right panels) or a detail (middle panel) of FMRP-stained primary tumours created after injection of control (left and middle panels) or *Fmr1*-silenced TS/A cells (right panel). The tumour margin is marked by a black arrow and also shown in the enlarged detail. (B) *Fmr1* mRNA expression levels in tumours generated with shRNA versus *Fmr1* shRNA (*n* = 5, *p* < 0.05, Student's *t*-test). (C) FMRP levels in normal and primary tumour tissues by Western blotting analysis. Lanes 1–3, protein extracts from breast tissues (mammary fat pad); lanes 4–11, 8 different murine breast tumours. FMRP levels were analysed by Western blot using specific FMRP antibodies (Ferrari et al, 2007) (left upper panel), the signal was normalized for Coomassie staining of the membrane (left lower panel). The right panel reports the quantification of the signals intensity (*p* < 0.01, Student's *t*-test). (D) Lung metastasis generated after orthotopic injection of control or *Fmr1* silenced TS/A cells were analysed for FMRP expression levels. Black arrows on left panels point to the metastasis, one of them is enlarged on the right panel. Scale bars: 200 μm (left panels) and 20 μm (right panels).

**Figure S5.** (A) Fmrp and GFP expression in 4T1 cells. 4T1 cells were transiently transfected using a recombinant plasmid containing the *Fmr1* shRNA (3 or 4) under the U6 promoter and the GFP in a separate transcription unit. Fmrp was detected by immunofluorescence. Scale bars: 20 μm. (B) Cancer cell survival in the bloodstream. Mice were injected intravenously (I.V.) with *Fmr1* silenced and control 4T1 cells, blood was collected 6 and 24 h after the injection (*n* = 5), and analysed as in panel B. (C) Lung metastasis formation in I.V. injected mice (*n* = 5).

**Figure S6.** (A) 4T1 cell morphology after Ca^2+^ deprivation. Upper panels: phase-contrast images of 4T1 cells expressing CTR shRNA and CTR vector after 18 min of EDTA treatment, lower panels: 4T1 cells expressing *Fmr1* shRNA and *GFP-FMR1*. All insets show time = 0. Scale bar: 50 μm. (B) Cell area in CTR and *Fmr1* shRNA cells (*n* = 18.590, *p* < 0.01) (C) Transendhotelial migration assay of CTR shRNA and *Fmr1* shRNA 4T1 cells (*n* = 5, *p* < 0.01). Histograms show the quantification of migrating cells. (D) 3D tumour cell spheroids invasion assay. 4T1 cells, stably transduced with CTR shRNA and *Fmr1* shRNA 4/5 and 3/5 (unpublished observations), were embedded in a collagene type I gel and imaged after 24 h. White dotted line indicates the spheroid body. Left panels indicate the quantification of the different parameters analysed (*n* = 15 per condition, *p* < 0.001, *p* < 0.001, Student's *t*-test). Scale bar = 200 μm.

**Figure S7.** FMRP immunoprecipitation from 4T1 cells with specific FMRP antibodies (Ferrari et al, 2007) and affinity purified pre-immune IgGs visualized by Western blotting.

**Figure S8.** Immunohistochemistry of FMRP, E-cadherin and Vimentin performed on human non-metastatic (BC) and metastatic (mBC) breast cancer (upper panels). Lower panel shows a correlation between FMRP, E-cadherin and Vimentin in BC and mBC (*n* = 14).

**Figure S9.** mRNA stability assay in 4T1 CTR and *Fmr1* shRNA cells. RNA was isolated at the indicated time points after Actinomycin D treatment and the stability of *Fn1*, *Jag1*, *Mmp9*, *Serpine1* and *Egfr* mRNAs was analysed by RT-qPCR (*n* = 5, * *p* < 0.05, ** *p* < 0.01).

**Figure S10.** Translational efficiency analysis in 4T1 CTR and Fmr1 shRNA cells. Quantification of the translational efficiency of Histone *H3.3, Mtap1b, Cav2, Dsp, Krt14* and *Mitf* mRNAs reported as ratio of P over NP (2^−[ΔCt(P)−ΔCt(NP)]^) (*n* = 4, * *p* < 0.05, ** *p* < 0.01).

**Figure S11.** (A) *E-cadherin* and *Vimentin* mRNA levels in 4T1 CTR and *Fmr1* siRNA (Ambion (ID 10919) cells detected by RT-qPCR (*n* = 3, *p* < 0.05). (B) Translational efficiency analysis in 4T1 CTR and *Fmr1* siRNA cells. Upper panel, polysome-mRNPs distribution on a sucrose gradient. Low left panel, fractions 1−5 corresponding to translating polysomes (P) and fractions 6−10 corresponding to silent mRNPs (NP) were pooled. Low right panel, quantification of the translational efficiency of *Histone H3.3*, *E-cadherin* and *Vimentin* mRNAs reported as ratio of P over NP (2^−[ΔCt(P)−ΔCt(NP)]^) (*n* = 3, *p* < 0.01).

**Table S1.**A TMAs used to detect FMRP expression

B FMRP expression on the TMA

**Table S2.** Clinical and pathological informations of the consecutive cohort of breast cancer patients (IFOM-Milan).

**Table S3.** Clinical and pathological informations of the breast cancer samples (UZLeuven).

**Table S4.**A Cancer occurring in the Cohort of 241 women with *FMR1* pre-mutation and full-mutation.

B Cancer incidence in the Cohort of 199 women with FMR1 pre-mutation and full-mutation

**Table S5.** FMRP target mRNAs in breast cancer cells.

**Table S6.** Clinical and pathological informations of the breast cancer samples (San Giovanni Rotondo Hospital).
