# The Fragile X Protein binds mRNAs involved in cancer progression and modulates metastasis formation

**DOI:** 10.1002/emmm.201470030

**Published:** 2014-04-02

**Authors:** Rossella Lucá, Michele Averna, Francesca Zalfa, Manuela Vecchi, Fabrizio Bianchi, Giorgio La Fata, Franca Del Nonno, Roberta Nardacci, Marco Bianchi, Paolo Nuciforo, Sebastian Munck, Paola Parrella, Rute Moura, Emanuela Signori, Robert Alston, Anna Kuchnio, Maria Giulia Farace, Vito Michele Fazio, Mauro Piacentini, Bart De Strooper, Tilmann Achsel, Giovanni Neri, Patrick Neven, D Gareth Evans, Peter Carmeliet, Massimiliano Mazzone, Claudia Bagni

**Correction to:** EMBO Mol Med (2013) 5, 1523–1536. DOI: 10.1002/emmm.201302847

The authors of the above research article regret two oversights.

First, one of the affiliations of the corresponding author, Claudia Bagni, was incorrect. The affiliations should read as 1, 2, 4 (see below).

(1) VIB Center for the Biology of Disease, Leuven, Belgium

(2) Center for Human Genetics, KU Leuven, Belgium

(4) Department of Biomedicine and Prevention, University “Tor Vergata”, Rome, Italy

Second, the control image for Vimentin shown in Fig 4B should be replaced by the one presented here.

**Figure 4B:**
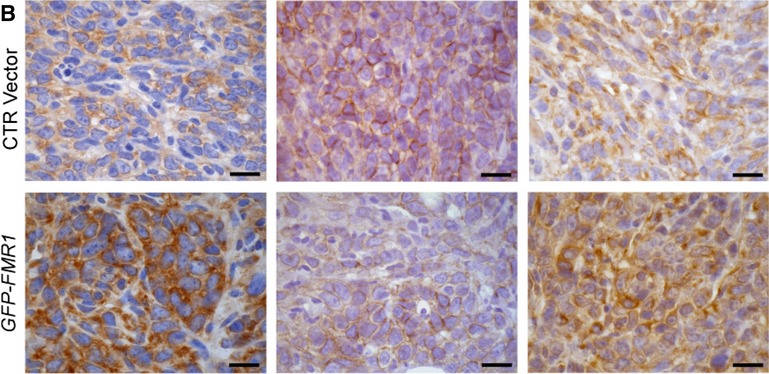


The results and conclusions of the article remain unchanged.

