# Supplementary material for: The Fragile X Protein binds mRNAs involved in cancer progression and modulates metastasis formation
Source: EMBO Mol Med. 2013 Sep 16;5(10):1523–36. doi: 10.1002/emmm.201302847 (PMC3799577; doi:10.1002/emmm.201302847)
Supplement: Supplementary file 1 [file emmm0005-1523-SD1.pdf]

EMBO Molecular Medicine

The Fragile X Protein binds mRNAs involved in cancer progression and modulates metastasis formation

Rossella Luca, Michele Averna, Francesca Zalfa, Manuela Vecchi, Fabrizio Bianchi, Giorgio La Fata, Franca del Nonno, Roberta Nardacci, Marco Bianchi, Paolo Nuciforo, Sebastian Munck, Paola Parrella, Rute Moura, Emanuela Signori, Robert Alston, Anna Kuchnio, Maria Giulia Farace, Vito Michele Fazio, Mauro Piacentini, Bart De Strooper, Tilmann Achsel, Giovanni Neri, Patrick Neven, D. Gareth Evans, Peter Carmeliet, Massimiliano Mazzone and Claudia Bagni

Corresponding author: Claudia Bagni, VIB Center for the Biology of Disease, Catholic University of Leuven

Review timeline:

Submission date:	03 April 2013
Editorial Decision:	03 May 2013
Revision received:	01 July 2013
Editorial Decision:	11 July 2013
Revision received:	01 August 2013
Accepted:	06 August 2013

Transaction Report:

(Note: With the exception of the correction of typographical or spelling errors that could be a source of ambiguity, letters and reports are not edited. The original formatting of letters and referee reports may not be reflected in this compilation.)

Editors: Céline Carret

1st Editorial Decision 03 May 2013

Thank you for the submission of your manuscript to EMBO Molecular Medicine and I apologize for the delay in getting back to you. We have now heard back from the three referees whom we asked to evaluate your manuscript. Although the referees find the study interesting and novel, they also raise a number of concerns that we would like you to address in a revised version of the study.

As you will see from the reports below, while referee 1 is enthusiastic about the study, the other two referees are more balanced. They would like to see better characterization of the correlation between FMRP expression and breast cancer molecular subtypes. Along with this line, better immunohistopathology data, including staining human tumors for Vimentin and E-cadherin would strengthen the clinical significance of the findings. Finally, but importantly, referee 3 requests additional data to provide some mechanistic insights and increase the overall impact of the study.

Given these evaluations, I would be happy to consider a revised version of your manuscript, with the understanding that the referees' concerns must be fully addressed and that acceptance of the manuscript would entail a second round of review.

Please note that it is EMBO Molecular Medicine policy to allow a single round of revision in order to avoid the delayed publication of research findings. Consequently, acceptance or rejection of the manuscript will depend on the completeness of your responses included in the next version of the manuscript.

EMBO Molecular Medicine has a "scooping protection" policy, whereby similar findings that are published by others during review or revision are not a criterion for rejection. Should you decide to submit a revised version, I do ask that you get in touch after three months if you have not completed it, to update us on the status.

Please also contact us as soon as possible if similar work is published elsewhere. If other work is published we may not be able to extend the revision period beyond three months.

I look forward to receiving your revised manuscript.

***** Reviewer's comments *****

Referee #1:

The manuscript "The Fragile X Protein binds mRNAs involved in cancer progression and modulates metastasis formation" is an excellent work on a very interesting medical genetics question: the relationship between FMR1 protein, which has a major role regulating the translation process in the polyribosomes, and cancer. Actually, this extensive and meticulous work includes a number of interesting questions and well achieved answers. Each of them could be an independent work, but the authors address well each result to the next question.
Particularly I have no major comments on the manuscript.
Comments minors:
1. - At the Introduction, authors mention that fragile X syndrome has an incidence of 1:2000 males and 1:4000 females (Bagni et al, 2012). Incidences currently handled for this syndrome are lower.
2.-On page 3 (Introduction) states that: "furthermore, a decreased risk of cancer has been Reported in patients with FXS (Rosales-Reynoso et al, 2010; Schultz-Pedersen et al, 2001). Actually Reynoso Rosales et al. did not report this risk diminished, they propose the Wnt7a gene as a candidate to explain the decreased risk of cancer in patients with fragile X syndrome.

Referee #3:

The ms by Luca et al describes a novel function of the fragile X mental retardation protein (FMRP) in cancer progression. In this work, the authors identified a high expression of the FMRP protein in a proportion of several tumor tissues, in particular in breast tumors where they found a correlation between high FMRP level and high tumor grade, high proliferation index and negative lymph node status. They then showed that a decrease or an increase of FMRP level within two breast cancer cell types (induced by shRNA or FMR1 transgene transfection) respectively modulates negatively or positively the metastatic properties of these cells in mouse as well as their invasion ability in vitro. Then the use of a crosslinking immunoprecipitation approach in one of the cell lines enabled the authors to identify 42 out of 84 tested mRNAs that are bound by FMRP. Among these targets vimentin and E-cadherin, important actors of cell adhesion, were validated as regulated targets of FMRP in mouse model of breast tumor (vimentin is upregulated by FMRP while E-cadherin is down regulated).
Although the direct medical impact of these new findings is maybe not so obvious, the quality of experiments performed by the authors is generally technically sound, their interpretation of the data are generally well supported by their experiments and the overall finding is of importance and of general interest. There are a few questions that could be important to address:

This study relies on the finding that a proportion of breast tumors have a higher FMRP expression than normal breast tissues. I feel that some precisions are needed to establish how this finding is significant. First, the description by authors of normal breast tissues as being FMRP negative is misleading. It is at least contradicting the fact that FMRP is well expressed in most tissues. Has breast tissue a lower FMRP expression than most tissues? Second, the proposed increase of FMRP

expression in tumor breast tissue could be due to an increase of cell density rather than a true increase of FMRP level per say. It is indeed stated that the increase of FMRP in breast tumor correlates with cell proliferation index and the pictures shown (e.g. fig 1 G) seem to show that tumorous tissues are denser than surrounding normal tissue. Thus, I wonder how unique this apparent increase of FMRP expression compares to the level of other cytoplasmic proteins in the same tissues? To clarify this issue it seems to me that it would be important to quantify this increase by western blot assays on the extracts of the tumor tissues to have a more quantitative view of this proposed FMRP level increase in comparison with other proteins.

The authors established some correlation between tumor grades and FMRP overexpression. For instance breast tumors with high grade (G3) show some FMRP overexpression in a higher proportion of cases (33.7%) than low grade tumors (19.5% for G1). However these data could be viewed as an indication that an increase of FMRP is not responsible for tumor aggressiveness since in 19.5% of the patients it has no impact? Do the multivariate Cox proportional hazard analyses take these negative numbers into consideration? Some explanations should maybe given for non cancer persons to emphasize the significance of the data and their potential medical importance.

The conclusion of the clip analysis presented in this work is that "FMRP acts as a master regulator of a large subset of mRNAs involved in multiple steps of cancer progression including invasion and intravasation". I disagree with this statement because it is not supported by actual data. First, the conclusion of the study is very likelely strongly biased by the choice of the 84 tested mRNAs. Second, the identification of 42 clipped mRNAs out of 84 tested indicates a very high non specific background (previous CLIP studies typically identified 1-3 % of total mRNAs) or a very low level of stringency. Third, the absence of presented data (only a list of mRNAs is given) prevents from any real appreciation of these results.

Fig4C : the actual western blot pictures (and not just their quantification) should be shown.

Supporting information: FMRP IP was performed using specific FMRP antibodies, it should be indicated more precisely which antibody was used for the assay.

This study could be improved by showing that E-cadherin and vimentin levels are also changed with FMRP on the human TMAs

Referee #4:

The manuscript submitted by Luca et al. reports that the Fragile X-encoded protein, FMRP, can play a role in tumor progression of locally advanced breast cancer patients, especially in those with a triple-negative subtype, and can correlate with a propensity for lung metastasis. In addition, the overexpression and depletion of FRMP in murine cell lines indicate its potential role in promoting or reducing metastasis, respectively. The authors provide evidence that FMRP drives EMT by binding to E-cadherin and Vimentin mRNA affecting translation and stability, respectively.
Figure 1B indicates that FMRP is somewhat more frequently expressed in grade 3 tumors; albeit, a significant number of positive grade 1 cases are found. Overall, FMRP is overexpressed in no more than 34% of grade 3 tumors. The puzzling finding is that a high expression FMRP correlates with node-negative patients known to be less proned for progression. How do the authors reconcile this with the findings of Figure 1E?
The micrographs in Figure 1C show that FMRP-expressing carcinoma cells do not necessarily localize at the edge of foci, and there is considerable heterogeneity in its expression level in different tumor foci. A low magnification micrograph would be mandatory to assess the distribution of FMRP.
The analysis of the breast cancer TCGA collection does not reveal an impressive difference in mRNA expression levels between triple-negative breast cancers, HER2- and ER/PgR-positive patients.
The experiments presented in Figures 2 and 3 use the prototypic highly metastatic murine cell line 4T1 and TS/A, a less metastatic cell line showing a reduction in lung metastasis in the FMRP shRNA-expressing cells. However, overexpressing cells are marginally showing an increase in lung

metastasis, particularly the TS/A cell line, which should have been expected to generate a higher metastatic index since the original cell line has a much lower propensity to metastasize than the 4T1 cell line.
FMRP binds to a number of RNA species, some of which were identified using a commercially available rtPCR array for 84 genes potentially involved in EMT. Then, the study focused on Vimentin and E-cadherin, two landmark genes of the epithelial and mesenchymal states, respectively. The study shows that FRMP controls E-cadherin and Vimentin by RNA translation and RNA stability, respectively, by two distinct mechanisms; these mechanisms would need to be further explored with other RNA species binding to FMRP and, in particular, binding to known EMT master gene candidates in order to address their relevance in tumor metastasis.
4T1 cells are certainly not appropriate to study such a mechanism, as these cells exhibit a rather mesenchymal-like phenotype. The authors need to consider a panel of cell lines with well-described E or M phenotypes. The function of Vimentin has remained elusive for many years, since no one has shown its implication in cell motility; albeit most, if not all, mesenchymal cells express Vimentin. Although the current findings may be potentially novel and lead to the exploitation of post-transcriptional mechanisms for the regulation of invasive and metastatic phenotypes, this study needs to provide much more convincing evidence if we are to believe that FMRP can play a role in the progression of carcinoma.

1st Revision - authors' response 01 July 2013

Point by Point

New experiments and images provided in the revised version:

1) FMRP expression level (Western blotting analysis) on a new set of health and matched breast cancer samples purchased from IMGENEX.
New Figure 1B

2) Immunohistochemistry for E-cadherin and Vimentin on non-metastatic and metastatic breast cancer using human breast samples described in Table S6.
New Supplementary Figure 8

3) mRNA stability assay for other five FMRP target mRNAs, key molecules involved in EMT.
New Supplementary Figure 9.

4) mRNA translational assay for other five FMRP target mRNAs, key molecules involved in EMT.
New Supplementary Figure 10

5) List of identified targets showing the fold change (enrichment) of the FMRP associated mRNAs.
Revised Table S5

6) Representative Western blotting referred to Figure 4C.
Revised Figure 4C

7) Western blotting analysis showing FMRP expression in different mouse tissues.
Figure 1 for the Referee only.

8) CDH1, CDH2 and Vimentin mRNA expression levels in the available breast cancer arrays (TCGA, 2012)
Figure 2 for the Referee only.

9) Immunohistochemistry for Her2 protein, localized at the plasma membrane as well as in the cytoplasm. This experiment was performed on the same TMA used to detect FMRP in Figure 1B.
Figure 3 for the Referee only.

10) Western blotting analysis for FMRP on 4 different human breast cancer cell lines
Figure 5 for the Referee only.

Referee #1:

The manuscript "The Fragile X Protein binds mRNAs involved in cancer progression and modulates metastasis formation" is an excellent work on a very interesting medical genetics question: the relationship between FMR1 protein, which has a major role regulating the translation process in the polyribosomes, and cancer. Actually, this extensive and meticulous work includes a number of interesting questions and well achieved answers. Each of them could be an independent work, but the authors address well each result to the next question.
Particularly I have no major comments on the manuscript.

We are delighted that the Referee appreciated our work.

Comments minors:
1.- At the Introduction, authors mention that fragile X syndrome has an incidence of 1:2000 males and 1:4000 females (Bagni et al, 2012). Incidences currently handled for this syndrome are lower.

We thank the Referee for his/her suggestion. The frequency in the population appears a bit different according to the published papers. Now it reads: with an estimated prevalence of approximately 1:2500 to 1:5000 in males and 1:4000-6000 in females. We have also added two more references reporting slightly different incidence (*Coffee et al., 2009 and Turk J, 2011).*

2.-On page 3 (Introduction) states that: "furthermore, a decreased risk of cancer has been Reported in patients with FXS (Rosales-Reynoso et al, 2010; Schultz-Pedersen et al, 2001). Actually Reynoso Rosales et al. did not report this risk diminished, they propose the Wnt7a gene as a candidate to explain the decreased risk of cancer in patients with fragile X syndrome.

We thank the Referee for this comment. Indeed the Schultz-Petersen referred to the decreased risk of cancer in FXS while Rosales-Reynoso referred to the expression of Wnt7a gene. We have modified the text accordingly.

Referee #3:

The ms by Luca et al describes a novel function of the fragile X mental retardation protein (FMRP) in cancer progression. In this work, the authors identified a high expression of the FMRP protein in a proportion of several tumor tissues, in particular in breast tumors where they found a correlation between high FMRP level and high tumor grade, high proliferation index and negative lymph node status. They then showed that a decrease or an increase of FMRP level within two breast cancer cell types (induced by shRNA or FMR1 transgene transfection) respectively modulates negatively or positively the metastatic properties of these cells in mouse as well as their invasion ability in vitro. Then the use of a crosslinking immunoprecipitation approach in one of the cell lines enabled the authors to identify 42 out of 84 tested mRNAs that are bound by FMRP. Among these targets vimentin and E-cadherin, important actors of cell adhesion, were validated as regulated targets of FMRP in mo use model of breast tumor (vimentin is upregulated by FMRP while E-cadherin is down regulated).
Although the direct medical impact of these new findings is maybe not so obvious, the quality of experiments performed by the authors is generally technically sound, their interpretation of the data are generally well supported by their experiments and the overall finding is of importance and of general interest. There are a few questions that could be important to address:
We thank the Referee for his/her positive comments on our work.
This study relies on the finding that a proportion of breast tumors have a higher FMRP expression than normal breast tissues. I feel that some precisions are needed to establish how this finding is significant. First, the description by authors of normal breast tissues as being FMRP negative is misleading. It is at least contradicting the fact that FMRP is well expressed in most tissues. Has breast tissue a lower FMRP expression than most tissues?

We agree with the Referee that the definition of "FMRP negative" in normal breast tissues is misleading. FMRP expression in normal breast tissues is indeed very low. The signal intensity varies between 0 and 1, with 0 being negative and 1 low expressed. We have now rephrased the text substituting "almost completely absent" with "expressed at lower levels".
Furthermore, we provide a Western blot showing mouse Fmrp expression in different tissues

(Figure 1 for the Referee only).

Second, the proposed increase of FMRP expression in tumor breast tissues could be due to an increase of cell density rather than a true increase of FMRP level per say. It is indeed stated that the increase of FMRP in breast tumor correlates with cell proliferation index and the pictures shown (e.g. fig 1 G) seem to show that tumorous tissues are denser than surrounding normal tissue.

We agree with the Referee that different cell density within tissue samples may complicate the assignment of the intensity score. However, immunohistochemistry is the ideal technique to obviate this inconvenience as it allows the detection of a given protein in situ, at the single cell level and shows exactly where it is located within the tissue examined (i.e. its cellular localization and cell type).
We performed a relative quantification of FMRP expression on tissue cores arrayed on TMA using IHC as described in the 'Materials and Methods' section and shown in Supporting Information Figure S1. Briefly, a semi-quantitative approach was applied to evaluate human FMRP expression by assigning the following intensity scores: 0, negative staining; 1, weak; 2, moderate; 3, intense. We hope the Reviewer can appreciate that despite the tumor cell density was comparable among the 4 selected representative breast tumor cases, the intensity of the FMRP staining ranged from 0 to 3 and, overall, quite homogeneous within each analyzed tumor core, thus indicating that FMRP expression was independent of cell density.

Thus, I wonder how unique this apparent increase of FMRP expression compares to the level of other cytoplasmic proteins in the same tissues?

We now provide the HER2 staining (membrane and/or cytoplasmic) on the same TMA analyzed for FMRP. HER2 displayed an intensity score ranging from 0 to 3 but did not parallel FMRP signal intensity on the same tumor samples, suggesting unique and exclusive expression of FMRP and HER2 (Figure 2 for the Referee only)
Since we have evaluated around 400 breast tumor samples we hope that the Referee is confident with our findings. In addition, we would like to mention that other targets have been analyzed (IHC and ISH) by the Molecular Pathology laboratory at the IFOM-IEO on the same TMA and described in (Nicassio et al., 2005. J Clin Invest.; Capra et al., 2006. Cancer Res, Vecchi et al., 2008. Oncogene; Vecchi et al., 2007. Oncogene, Confalonieri et al., 2009. Oncogene, Colaluca et al., 2008. Nature, Amson et al., 2011. Nat Med.).

To clarify this issue it seems to me that it would be important to quantify this increase by western blot assays on the extracts of the tumor tissues to have a more quantitative view of this proposed FMRP level increase in comparison with other proteins.

We agree with the reviewer that Western blotting (WB) analysis could provide an independent quantification of FMRP expression in breast cancer, however, frozen sections from the same tissues to be used for Western blotting analysis are not available. In order to overcome this limitation and follow the Referee suggestion we performed a WB analysis on a new set of commercially available ductal breast cancer samples (stage 2-4) in which we monitored FMRP as well the cytoplasmic protein a-Tubulin (that is not among the FMRP targets). These data have been now provided as New Figure 1B.

The authors established some correlation between tumor grades and FMRP overexpression. For instance breast tumors with high grade (G3) show some FMRP overexpression in a higher proportion of cases (33.7%) than low grade tumors (19.5% for G1). However these data could be viewed as an indication that an increase of FMRP is not responsible for tumor aggressiveness since in 19.5% of the patients it has no impact?

Cancer progression is a multistep process that requires involvement of several biological mechanisms and sequential acquisition of multiple genetic lesions. Therefore, we presume that FMRP may cooperate with other genes to ultimately determine cancer metastatic spreading. This would explain why there might be a fraction of tumors positive to FMRP but with a less aggressive characteristics (i.e. G1 tumors).
What is clear from our data is that overexpression of FMRP is a hallmark of enhanced tumor aggressiveness; indeed it is more probable that a tumor acquires more aggressive features (high-

grade/high-proliferation) when FMRP is overexpressed or, vice versa, it is less probable that a more differentiated tumor does overexpress FMRP. Moreover, we provide evidences that FMRP mRNA targets are involved in EMT transition, invasion and intravasation and, as such, determinant for cancer progression and metastatic process, which highlight again a relevant role of FMRP in tumor aggressiveness.

Do the multivariate Cox proportional hazard analyses take these negative numbers into consideration? Some explanations should maybe given for non cancer persons to emphasize the significance of the data and their potential medical importance.

We thank the Referee for this suggestion; we have now explained differently the meaning of these findings. We performed a multivariate analysis using a Cox model in order to check whether the levels of FMR1 expression independently correlate with the propensity of breast tumor cells to metastasize to lungs. Significant p-values indicates that high FMR1 expression indeed correlates with lung metastases and independently to the estrogen receptor status that was the only available clinical parameter for all the datasets considered in the analysis.
In order to explain better this result we now modified the main text as follows: "Cox proportional hazard analysis of the three cohorts revealed that patients with FMR1 overexpressing breast tumors have an increased risk to develop lung metastasis (Hazard Ratio = 1.21; 95% CI 1.02-1.45, p = 0.0293) and this is independent to estrogen receptor status (HR = 1.51 95% CI 1.27-1.85, p<0.0001), the only pathological parameter available for all datasets considered. This suggests that FMRP increased expression might have a role in metastatic spreading of breast tumor cells to the lungs.".

The conclusion of the clip analysis presented in this work is that "FMRP acts as a master regulator of a large subset of mRNAs involved in multiple steps of cancer progression including invasion and intravasation". I disagree with this statement because it is not supported by actual data. First, the conclusion of the study is very likelely strongly biased by the choice of the 84 tested mRNAs. Second, the identification of 42 clipped mRNAs out of 84 tested indicates a very high non-specific background (previous CLIP studies typically identified 1-3 % of total mRNAs) or a very low level of stringency. Third, the absence of presented data (only a list of mRNAs is given) prevents from any real appreciation of these results.

We thank the Referee for this comment. According to the large studies published over the last 10 years, FMRP targets account for 4-27% of the cellular transcriptome (Aschley et a., 1993, Brown et al., 2001, Darnell et al., 2011, Ascano et al 2012). However the list of targets varies according to the biological material, threshold and technique used. In our study we show that FMRP affects cell intravasation and invasion (Figures 2F and Figure 3) and this was the rationale to use a specific Array, including molecules involved in these biological functions, to identify FMRP targets. Since we have used a preselected plate that matched the observed phenotype this could explain why 50% of the genes tested are targets of FMRP.
We have now extended our studies and provide further information on; 1) the fold change (enrichment) of the FMRP associated mRNAs compared to control. We considered as FMRP targets only the ones with a fold change higher than 7 respects to the negative control (IgGs). We would like to mention that these experiments were performed by independent collaborators and qPCR devices (n=3), 2) stability assay for 5 more mRNA targets, 3) translational assay for 5 more mRNA targets.
These findings are now shown in Revised Supporting information Table S5, New Supplementary Figure 9 and 10.

Fig4C: the actual western blot pictures (and not just their quantification) should be shown.

We have now provided the Western blot pictures in the revised Figure 4C.

Supporting information: FMRP IP was performed using specific FMRP antibodies, it should be indicated more precisely which antibody was used for the assay.

All the experiments described in the manuscript have been performed using a well-characterized antibody (Supplementary Figure 1 and Ferrari et al 2007; Napoli et al., 2008). The antibody is very efficient in immunoprecipitation, IHC and western blotting experiments. We have added an

additional sentence in the revised text as well.

This study could be improved by showing that E-cadherin and vimentin levels are also changed with FMRP on the human TMAs

As previously discussed we do not have access to the same TMA used to evaluate FMRP (Supporting information Table S2). To follow the Referee suggestion we have used primary tumors samples of non-metastatic (BC) and metastatic (mBC) breast cancer described in Table S6 and show a direct correlation between FMRP and Vimentin and an inverse correlation between FMRP and E-cadherin.
New Supplementary Figure 8.

Referee #4:
The manuscript submitted by Luca et al. reports that the Fragile X-encoded protein, FMRP, can play a role in tumor progression of locally advanced breast cancer patients, especially in those with a triple-negative subtype, and can correlate with a propensity for lung metastasis. In addition, the overexpression and depletion of FRMP in murine cell lines indicate its potential role in promoting or reducing metastasis, respectively. The authors provide evidence that FMRP drives EMT by binding to E-cadherin and Vimentin mRNA affecting translation and stability, respectively.
Figure 1B indicates that FMRP is somewhat more frequently expressed in grade 3 tumors; albeit, a significant number of positive grade 1 cases are found. Overall, FMRP is overexpressed in no more than 34% of grade 3 tumors. The puzzling finding is that a high expression FMRP correlates with node-negative patients known to be less proned for progression. How do the authors reconcile this with the findings of Figure 1E?

We thank the Referee for this comment. As we have already discussed in the answer to Referee 3 the fraction of tumors positive to FMRP but with a less aggressive characteristics (i.e. G1 tumors) could be explained because during cancer progression several biological mechanisms are needed as well as acquisition of multiple genetic lesions. Therefore, we cannot exclude that FMRP may cooperate with other genes to ultimately determine cancer metastatic spreading.
Additionally to the axillary lymph node involvement, tumour diameter and histological grade *have to be considered for the prognosis of breast cancer (Harris JR, 2004). In agreement, FMR1 mRNA expression was increased with a high statistical significance (p-value = 0.0007) in the more aggressive triple negative breast cancer (TNBC) subtype (i.e. ER/PgR and HER2 negative) compared to the ER/PgR and/or the HER2 positive tumours in the large cohort of breast cancer patients (597 samples) recently made available by the Tumor Cancer Genome Atlas consortium (TCGA) (see Fig. 1F).*
We have now expanded on this point and discuss that tumor like TNBC are heterogeneous, some are node-negative while others are node-positive. Both categories are very aggressive. 20-30% of the node-negative will relapse within 5 years (very aggressive) according to Ueno et al 2011 and are more likely to metastasize to lung without involvement of LN as reported in Brouckaert et al., 2012 and in Van Belle et al., 2009.

The micrographs in Figure 1C show that FMRP-expressing carcinoma cells do not necessarily localize at the edge of foci, and there is considerable heterogeneity in its expression level in different tumor foci. A low magnification micrograph would be mandatory to assess the distribution of FMRP.

We agree with the Referee that the distribution of FMRP in Figure 1C is heterogeneous from high to very high, this is also in agreement with the heterogeneity of the tumor cells. We have now changed the text commenting on the heterogeneity of FMRP expression in different tumor foci that is also visible in Supplementary Figure 1.

The analysis of the breast cancer TCGA collection does not reveal an impressive difference in mRNA expression levels between triple-negative breast cancers, HER2- and ER/PgR-positive patients.

We have now included the expression analysis of a couple of prototypical molecular markers for EMT, i.e. E-cadherin (CDH1), N-cadherin (CDH2) and Vimentin (VIM), on the same large cohort

of breast cancer patients analyzed for FMR1 in Figure 1F (made available by the Tumor Cancer Genome Atlas consortium, on a total of 597 samples). These EMT transcripts analyzed were confirmed to change in the more aggressive triple negative breast cancer (TNBC) subtype compared to the ER/PgR or HER2 positive tumours (Figure 3 for the Referee only).

The experiments presented in Figures 2 and 3 use the prototypic highly metastatic murine cell line 4T1 and TS/A, a less metastatic cell line showing a reduction in lung metastasis in the FMRP shRNA-expressing cells. However, overexpressing cells are marginally showing an increase in lung metastasis, particularly the TS/A cell line, which should have been expected to generate a higher metastatic index since the original cell line has a much lower propensity to metastasize than the 4T1 cell line.

As the Referee has correctly pointed out, the TS/A cell line overexpressing FMRP increases its metastatic potential by 70% while the 4T1 (already highly metastatic) by 50%. This indicates that FMRP overexpression increases the metastatic potential of the TS/A cell line more than in the 4T1 cell line. We have now added a sentence in the revised version to better explain these findings.

FMRP binds to a number of RNA species, some of which were identified using a commercially available rtPCR array for 84 genes potentially involved in EMT. Then, the study focused on Vimentin and E-cadherin, two landmark genes of the epithelial and mesenchymal states, respectively. The study shows that FRMP controls E-cadherin and Vimentin by RNA translation and RNA stability, respectively, by two distinct mechanisms; these mechanisms would need to be further explored with other RNA species binding to FMRP and, in particular, binding to known EMT master gene candidates in order to address their relevance in tumor metastasis.

We have followed the Referee's suggestion and, in addition to E-cadherin and Vimentin, we analyzed mRNA translation as well mRNA stability of ten other identified FMRP targets. New Supplementary Figure 9 and 10.

4T1 cells are certainly not appropriate to study such a mechanism, as these cells exhibit a rather mesenchymal-like phenotype.

We have based our studies on two cell types (4T1 and TSA). The choice of the 4T1 mouse mammary tumor cell line was dictated by the capacity to efficiently metastasize (Tao et al., 2008). Moreover, these cells show an epithelial cellular morphology and express markers such as E-cadherin rather than N-cadherin (mesenchimal marker).

The authors need to consider a panel of cell lines with well-described E or M phenotypes. The function of Vimentin has remained elusive for many years, since no one has shown its implication in cell motility; albeit most, if not all, mesenchymal cells express Vimentin.

We agree with the Referee that the involvement of Vimentin was under debate in the past, but we also feel that there is quite a massive number of publications pointing to a key effect of Vimentin on the number of circulating tumor cells and their metastatic potential, just to mention a few of them 1) Vimentin expression increases during the EMT associated with tumor invasion and metastasis, especially among breast cancers and melanomas. Vimentin predicts poor breast cancer survival (Korsching et al. 2005) and high incidence of circulating tumor cells with high levels of Vimentin have been detected in patients with metastatic disease compared to early stage breast cancer (Callergi et al., Breast Cancer Research 2011). This again, strongly supports the notion that EMT is involved in the metastatic potential of CTCs and that Vimentin is contributing to the metastatic phenotype. 2) Willipinski-Stapelfeldt et al. [2005] studied Vimentin expression in a very large cohort of breast cancers (n = 2,517) They showed that Vimentin expression in tumour cells correlated significantly with high tumour grade, high mitotic index and negative estrogen/progesterone receptor status (Willipinski-Stapelfeldt, B.,et al Clin Cancer Res 2005) 3) Vimentin is proposed as target for breast cancer metastasis and in a panel of soft tissue sarcoma (Thaiparambil et al Cancer research 2009; Lahat et al PLoS One 2010)61) it has been reported that Vimentin nicely correlates with cells that underwent EMT (Mani et al., Cell 2008).

Although the current findings may be potentially novel and lead to the exploitation of post-transcriptional mechanisms for the regulation of invasive and metastatic phenotypes, this study

needs to provide much more convincing evidence if we are to believe that FMRP can play a role in the progression of carcinoma.

Following the Referee's suggestion we have now analyzed FMRP expression in a panel of human breast cancer cell lines with epithelial and mesenchimal characteristics and provided this information as Figure 4 for the Referee only.
We have also extended the molecular analysis of FMRP mediated posttranscriptional regulation to other 10 mRNA targets and provide these findings in two new New Supplementary Figures 9 and 10. Furthermore our in vivo data show, using two different cell lines with different metastatic potential that FMRP affects metastasis formation, complementing our cellular and molecular findings. Last but not least we were also able to monitor cancer incidence in a cohort of patients with FXS (with absent or reduced FMRP) and show that they have a decreased cancer incidence. All together we have provided multiple and complementary evidence that we hope convince the Referee on the importance of our findings.

2nd Editorial Decision 11 July 2013

Thank you for the submission of your revised manuscript to EMBO Molecular Medicine. We have now received the enclosed report from the referee who was asked to re-assess it. As you will see the reviewer is now supportive and I am pleased to inform you that we will be able to accept your manuscript pending the following final amendments:

-Please carefully proof-read the manuscript for typos as suggested by the referee. In addition, we would appreciate if you could reflect the referee's concern with a line or two in the discussion section.
"Contrary to their statement Vimentin has still today a very elusive function in invasive migration, albeit it is routinely used as a mesenchymal marker."

And on an editorial note:

-Please upload the final high-resolution Supplementary Information single pdf file, including on its 1st page the title, authors and a Table of Content (see below).

- Please provide within the manuscript Authors Contribution, The Paper Explained and possibly For More Information (see below).

-Please remove all red text within the body of the manuscript.

-For human tissue collection and animal experiments, please rephrase and complete the material and method section as indicated in our Guidelines:
http://onlinelibrary.wiley.com/journal/10.1002/(ISSN)1757-4684/homepage/ForAuthors.html#use

Please submit your revised manuscript within two weeks.

I look forward to reading a new revised version of your manuscript as soon as possible.

***** Reviewer's comments *****

Referee #4 (Remarks):

The manuscript has been extensively revised with new sets of more convincing data.
The authors should proof-read the manuscript for typos such as mesenchimal!
Contrary to their statement Vimentin has still today a very elusive function in invasive migration, albeit it is routinely used as a mesenchymal marker.

2nd Revision - authors' response 01 August 2013

I am submitting the revised manuscript entitled "The Fragile X Protein binds mRNAs involved in cancer progression and modulates metastasis formation"
We have modified the text and the Figures according to the Journal requirements and:
1) added the Paper Explained;
2) added the Authors contribution;
3) added in the discussion the following sentence "Although *Vimentin* has still an elusive function in invasive migration it is used as a mesenchymal marker" as suggested by the Referee;
4) Modified the Point by Point. We have removed the four Figures that were included for the

Thank you very much for your time and consideration of our work.
