# Supplementary material for: The Fragile X Protein binds mRNAs involved in cancer progression and modulates metastasis formation
Source: EMBO Mol Med. 2013 Sep 16;5(10):1523–36. doi: 10.1002/emmm.201302847 (PMC3799577; doi:10.1002/emmm.201302847)
Supplement: Supplementary file 2 [file emmm0005-1523-SD2.pdf]

Luca et al. Supporting Information

The Fragile X Protein binds mRNAs involved in cancer progression and modulates metastasis formation

Table of Contents

- **Materials and Methods**
- **Figure Legends S1-S11**
- **References**
- **Figures (S1-S11)**
- **Tables (S1-S6)**

Materials and Methods

Patients Information. Patients from the European Institute of Oncology (Milan, Italy, Supplementary Tables S1 and S2).

The histopathological diagnoses of the tumours were described according to the World Health Organization (WHO) International Classification of Disease for Oncology. The clinical staging was determined by the TNM Staging System and the Elston and Ellis grading System. The malignancy of infiltrating carcinomas was scored according to the Scarff-Bloom-Richardson classification.

Some of the specimens used in Fig. 1D were provided by the Molecular Pathology Unit at the European Institute of Oncology (IFOM-IEO, Milan, Italy). All human tissues were collected following standardized procedures including and informed consent (see main text).

Each sample was histopathologically evaluated to ensure the presence of at least 80% of tumour cells. The medical records of all patients were examined to obtain clinical and histopathological information.

Tissue MicroArrays Generation

Normal and tumour samples, formalin-fixed and paraffin-embedded (FFPE), were provided by the Pathology Departments of Ospedale Maggiore (Novara, Italy), Presidio Ospedaliero (Vimercate, Italy), Ospedale San Paolo (Milan, Italy) and Ospedale Sacco (Milano) and processed for the analysis of FMRP expression on TMAs (as described in Supporting Information Table S1). In colorectal, lung, prostate, and bladder tumours (T), the normal (N) samples were derived from the same patients whenever possible. In breast tumours, the normal samples were fibroadenomas and, when available, paired normal counterparts (as specified in Supporting Information Table S1). Samples were arrayed in four different TMAs (details in Supporting Information Table S1) as previously described (Capra et al, 2006; Kononen et al, 1998). Briefly, two representative normal and tumour areas (diameter 0.6 mm) from each sample, previously identified on hematoxylin–eosin-stained sections, were removed from the donor blocks and deposited on the recipient block using a custom-built precision instrument (Tissue Arrayer-Beecher Instruments).

Immunohistochemical analysis of FMRP on human tissues.

For the FMRP study in human breast cancer and correlation analysis with clinically relevant parameters, we used data from a cohort of breast cancer patients enrolled in a surgical trial

conducted at the European Institute of Oncology (IEO) between March 1998 and December 1999 (Veronesi et al, 2003), involving 477 consecutive patients (median age 55.8, range 37-75 years) with small-size primary breast cancers (pT1 and pT2<3.0 cm in diameter) and a mean follow-up period of 65 months. The overall clinical and pathological characteristics are given in Supporting Information Table S3 and previously described (Confalonieri et al, 2009). Immunohistochemical analysis of FMRP was performed by tissue microarray. Formalin-fixed, paraffin-embedded tumour blocks were retrieved from the Pathology Department of the IEO and arrayed on different TMAs (Confalonieri et al, 2009). For each patient, 2 representative cores were arrayed. TMA blocks were cut in 2 μm sections, mounted on glass slides and processed for IHC.

Estrogen and progesterone receptors, Ki67 and ErbB2 or HER2/neu, evaluated by IHC on whole tissue sections, were retrieved from histopathologic reports. ErbB2 or HER2/neu overexpression was evaluated according to the FDA-approved scoring system recommended by the DAKO Hercep Test. FMRP IHC was performed using polyclonal antibodies (Ferrari et al, 2007) (1:500 dilution), followed by detection with the EnVision Plus/HRP detection system (DAKO). A semi-quantitative approach was used to evaluate FMRP protein expression, scored as follows: 0, negative staining; 1, weak-moderate, 2, moderate-intense; 3, very intense. Samples were divided into negative and positive according to an FMRP IHC score ≤ 1.0 or > 1.0, respectively. FMRP expression was positive at least in 80-90% of the tumour tissue analyzed (including samples from UZ Leuven).

Assessment of FMRP expression (Fig. 1A, C): IHC signal was associated with the normal and tumour cell component, and not with the adjacent or infiltrating stroma. Those cores in which the epithelial component was absent were discarded from the analysis. Assessment of FMRP expression (Fig.1H): samples (tumour and matched lung metastasis, n = 12) were processed at the same time and evaluated by a pathologist using a light microscope, without no knowledge of either the clinical or histological diagnosis, and according to the scoring system described above.

TMA IHC data were analyzed using JMP IN 5.0 software (SAS). A p-value of less than 0.05 was considered as significant.

Kaplan Mayer curves

The probability of having lung metastasis due to *FMR1* mRNA overexpression was calculated as follows: we used as cut-off the 75th percentile of the distribution of *FMR1* expression $\log_2$ values across all patients in every cohort analyzed (i.e., EMC-344, MSK-99 and NKI-295). Patients were then stratified in high- (>75th) or low (≤75th) *FMR1* expression, and Kaplan-Meier curves with log-rank test were performed to calculate the relative lung metastasis probability in the two

groups. Consequently, a computer algorithm was used for patients' stratification: the experimenter was not involved in the decision to classify a patient in the high- or low-*FMR1* expression groups.

Patients from the University Hospital Leuven (Belgium, Supporting information Table S3)

Some of the specimens used in Fig.1H were provided by the University Hospital Leuven and samples collected according to a standardized method. Histopathologic examination was performed on hematoxylin and eosin–stained sections and evaluated to ensure the presence of at least 80% of tumour cells. Tumours were classified and graded according to the WHO Classification and the Elston and Ellis grading system, respectively.

Mice and Animal Care

Animal care was conducted conforming to the institutional guidelines that are in compliance with Italian laws (DL N116, GU, suppl 40, 18-2-1992), international laws and policies (European Community Council Directive 86/609, OJa L 358, 1, December 12, 1987; National Institutes of Health Guide for the Care and Use of Laboratory Animals, US National Research Council, 1996) and approved by the Institutional Ethical Board at the Katholieke Universiteit of Leuven, Belgium. Nine weeks old Balb/c female mice were used in this study.

Silencing and overexpression of *Fmr1* mRNA using a lentiviral vector

To silence *Fmr1* mRNA, five independent shRNAs plasmids (# SHCLND-NM_008031 Sigma-Aldrich) were tested.

1) shRNA #1: targeting *Fmr1* 3'UTR

5'-CCGGCCTTACATAAACATCAGCTTACTCGAGTAAGCTGATGTTTATGTAAGGTTTTTG-3'

2) shRNA #2: targeting *Fmr1* CDS

5'-CCGGCGCACCAAGTTGTCTCTTATACTCGAGTATAAGAGACAACTTGGTGCGTTTTTG-3'

3) shRNA #3: targeting *Fmr1* CDS

5'-CCGGCCACGAAACTTAGTAGGCAAACTCGAGTTTGCCTACTAAGTTTCGTGGTTTTTG-3′;

4) shRNA #4: targeting *Fmr1* CDS

5′- CCGGCCACCACCAAATCGTACAGATCTCGAGATCTGTACGATTTGGTGGTGGTTTTTG-3′;

5) shRNA #5: targeting *Fmr1* CDS

5′- CCGGGAGGATGATAAAGGGTGAGTTCTCGAGAACTCACCCTTTATCATCCTCTTTTTG-3′.

6) Control scramble shRNA (#SHC002 Sigma-Aldrich)

5'-CCGGCAACAAGATGAAGAGCACCAACTCGAGTTGGTGCTCTTCATCTTGTTGTTTTT-3'

shRNA plasmids were transiently transfected (48hrs) in 4T1 cells using lipofectamine (Supporting Information Fig. S3B). Only shRNAs #3, #4, #5 were then used to generate lentiviral particles to silence FMRP in tumour cells. For all in vivo experiments we used a combination of shRNAs #3 and #4 to infect 4T1 or TS/A cell lines. For the 3D tumour spheroids invasion assay we also used other two combinations of shRNAs (shRNA #4/5 and shRNA #3/5).

Second generation plasmids (Naldini et al, 1996) were used to generate transduction particles using HEK293T as packaging cells by calcium phosphate transfection method (Chen & Okayama, 1987). Virus-containing supernatants from HEK293T cells were collected after 24 hrs and filtered through 0.22 mm filters (Millipore). Cells (4T1 or TS/A) were infected with two different lentiviruses carrying specific shRNA (#3, #4, #5) in presence of 8 mg ml^{-1} polybrene (Sigma) and incubated overnight. Infection efficiency was checked by parallel infection with GFP lentivirus. The parental vector (pLKO.1-puro) (Sigma-Aldrich) allows monitoring stable transfection via puromycin resistance selection. The virus has been propagated in episomal form and kept at -80°C. The silencing efficiency was verified by Western blot analysis (Supporting Information Fig. S3B-D).

4T1 cells were also infected with lentiviral particles carrying a GFP *Fmr1* shRNA construct. GFP coding region and *Fmr1* shRNAs (#3 or #4) or CTR shRNA (Sigma) were cloned under the control of independent promoters.

Finally, overexpression of *hFMR1* mRNA was obtained using the lentiviral vector (207.pRRLsyn.PPTs.hCMV.GFP.Wpre) kindly provided by Prof. Suzanne Zukin (Albert Einstein University, USA).

Immunofluorescence

4T1 GFP-CTR and GFP-*Fmr1* shRNA cells were fixed with PFA 4% for 20 min and stained 2 hrs at room temperature with FMRP (Ferrari et al, 2007) (1:1000) and E-cadherin (1:200 BD) antibodies. Cells were then incubated with Cy3 and Cy5 anti-rabbit antibodies (1:1000, Amersham) and mounted using Fluoromount. Images were acquired using an Olympus microscope with a 60X oil objective. Control stainings were performed omitting primary antibodies.

Silencing of *Fmr1* mRNA using siRNAs

siRNAs specific for *Fmr1* mRNA full length were purchased from Ambion (ID #11010 and

#10919). Silencing of 4T1 cells was performed using Lipofectamin2000 (InVitrogen) according to manufacturer's instructions.

Western blot

Cells and tumours were lysed in 100 mM NaCl, 10 mM $MgCl_2$, 10 mM Tris-HCl pH 7.5, 1% Triton X-100, 1 mM DTT, 40 U ml^{-1} RNAse OUT (Invitrogen), 5 mM β-glycerophosphate, 0,5 mM Na_3VO_4, 10 μl ml^{-1} Protease inhibitor cocktail (PIC, Sigma) or 50mM Tris HCl pH7,4, 150mM NaCl, 1%, DOC, 1% NP-40, 10ml ml^{-1} PIC. After 5 min of incubation on ice, the lysates were centrifuged 15 min at 16,000g at 4°C. 10-20 μg of supernatant were separated by SDS–PAGE electrophoresis and transferred to a PVDF membrane (Millipore). Membranes were incubated using specific antibodies for FMRP (Ferrari et al, 2007) (1:1000), mouse Vinculin (1:2000, Sigma-Aldrich), mouse E-cadherin (1:500, BD Bioscience), mouse GAPDH (1:20000, Chemicon), rabbit rpS6 (1:1000, Cell Signaling), mouse Vimentin (1;1000 SIGMA), mouse a-Tubulin (1: 1000, Hybridoma Bank) and signal was detected using an enhanced chemiluminescence kit (GE Healthcare). Membranes were then stained with Coomassie.

FMRP and a-Tubulin were detected on INSTA-Blot™ Breast Tissue OncoPair (Cat IMB-130e from Imgenex) using specific antibodies for FMRP (Ferrari et al, 2007)(1:1000) and mouse a-Tubulin (1: 1000, Hybridoma Bank). Proteins were normalized by the amido-black staining.

Intravasation assay

$1X10^6$ GFP labelled 4T1 cells resuspended in 30μl D-PBS were injected in the right second thoracic mammary fat pad of 9 weeks old Balb/c female mice. 29 days after injection 400 μl of blood were collected by retro-orbital bleeding and subjected to haemolysis and RNA extraction using QIAamp RNA Blood Mini Kit (QIAGEN) following the manufacturer's instruction. 500 ng of RNA was subject to retrotranscripition and used to amplify GFP mRNA in a LightCycler 480 apparatus (Roche Diagnostics). The reaction mixtures contained the cDNA and the LightCycler-DNA Master SYBR Green I Buffer (Roche) and *GFP* specific primers (fw: 5'-TACAACAGCCACAACGTCTAT-3' and rev: 5'-GGATCTTGAAGTTCACCTTGATG-3') or *Histone H3.3* (fw: 5'-ACTGACTGTTCACAGACC-3' and rev: 5'-CCATCCCTTCTGCGTATTAG-3') used for normalization. mRNA levels were calculated as follows: $2^{-[\Delta Ct(sample) - \Delta Ct(Average\ CTR)]} = 2^{-\Delta\Delta Ct}$, where ΔCt equals Ct (GFP) -Ct (*Histone H3.3*). For each mRNA, a standard curve was prepared using serial dilutions.

Cell survival and extravasation assay

8 x 10^4 GFP labelled cells resuspendend in 200μl D-PBS were injected in the tail vein of 9 weeks old Balb/c female mice. 400μl of blood were collected by retro-orbital bleeding at 6 and 24 hours after injection and subjected to haemolysis and RNA extraction using QIAamp RNA Blood Mini Kit (QIAGEN) following the manufacturer's instruction. RT-qPCR was performed to analyse *GFP* RNA levels using LightCycler apparatus (Roche Diagnostics), as described above. mRNA levels were calculated as follows: $2^{-[\Delta Ct(time\ point) - \Delta Ct(time\ 0)]} = 2^{-\Delta\Delta Ct}$, where ΔCt equals Ct (GFP) -Ct (*Histone H3.3*). Finally, an independent group of mice was sacrificed 10 days after injection, lung metastasis were stained with Indian ink and counted under a dissecting microscope.

Multicellular Tumour Spheroids (MCTS) three-dimensional invasion assay

4T1 confluent cultures were trypsinized, washed in D-PBS and resuspended in DMEM-F12. 5 x 10^3 cells/spheroid were suspended in 20% methylcellulose in DMEM-F12 plus EGF. Drops of the cell suspension were placed onto the lid of 150 mm dish, which was then flipped over the dish. Hanging drop cultures were incubated for 24 hrs and the resulting cellular aggregates were washed and recovered with a large pipette tip and harvested according to (Hattermann et al, 2011). Spheroids were implanted into a three-dimensional collagen gel adapted from (Del Duca et al, 2004). Briefly, a collagen solution was prepared consisting of 1.5 mg/ml collagen type I (Millipore, rat tail), 2 mg/ml bicarbonate in methylcellulose plus 40% FBS and pH controlled. Five hundred microliters of this solution was added to 24-well plates and spheroids were implanted into the gel using a large pipette tip. After gelation at 37°C in a humidified atmosphere of 5% CO_2 for 20 min, the gel was overlaid with 500 µl of DMEM-F12 supplemented with EGF. Tumour cell spheroids were fixed at 24hrs with 4% PFA and cell invasion was evaluated using the Image J software. Invading area was calculated as follows: total area minus body area; protrusion number: average of the protrusion number over body perimeter. These parameters were analyzed in 20 independent spheroids per condition. The experiment was performed in triplicates.

Transendothelial migration assay

HUVEC (1 x 10^5) were plated onto the upper side of a 6.5 mm diameter, 8 μm pore filter transwell (Corning) previously coated with Fibronectin (20 μg/ml) and incubated with complete growth medium for 24 hrs at 37°C. HUVEC were next activated with 5ng/ml of IL-1 for 4hrs. 5 x 10^4 cancer cells (4T1 CTR shRNA and 4T1 *Fmr1* shRNA) were seeded in the upper chamber and DMEM supplemented with 20% con FBS was added in the lower compartment. Cells were let to

migrate for 18 hrs at 37°C, washed with PBS and fixed with 4% paraformaldehyde. Cells on the upper membrane were removed with a cotton swab and membranes stained with 0,1% Crystal Violet for 20 min. Inserts were washed with MilliQ water and air-dried. Picture of each membrane was taken using a dissecting microscope and the number of transmigrated cells was counted.

EDTA adhesion assay

4T1 CTR, *Fmr1* shRNA, *GFP-FMR1* and empty vector cells were plated on plastic and grown to 80% confluence for 48 hrs. Next, 0.5 mM EDTA was added in order to chelate calcium. Changes in the morphology of the cells were recorded by live imaging for 18 min using the IN Cell Analyzer 2000 (GE Healthcare).

Cell area analysis

Control and FMRP silenced cells were plated at a low density on a 24 multiwell plate. After 10 hrs, cells were fixed in 4% PFA for 20 min at room temperature and stained for nuclei (DAPI), F-actin (TRITC-Phalloidin, Sigma) and FMRP (Ferrari et al, 2007)(1:1000). To investigate the cell spreading, cells were consequently imaged using the IN Cell Analyzer 2000 (GE Healthcare). Individual cells were identified on the basis of the nuclear staining and the cell shape was revealed and analysed using Phalloidin staining. A custom made protocol (Freeley et al, 2010) was established using the IN Cell Analyzer-workstation multi-target-analysis to measure the cell area. Quantitative analysis based on single cell measurements is shown for a total of 18.590 cells.

Immunoprecipitation followed by Western blotting

Lysis of mouse breast tumours or cells was carried out on ice in 100 mM NaCl, 10 mM $MgCl_2$, 10 mM Tris-HCl pH 7.5, 1% Triton X-100, 1 mM DTT, 400 U ml^{-1} RNAse OUT (Invitrogen), 10 μl ml^{-1} Protease inhibitor cocktail (Sigma-Aldrich), 5 mM β-glycerophosphate, 0,5 mM Na_3VO_4. After 5 min of incubation on ice, lysates were centrifuged for 5 min at 12,000g at 4°C and 500 μg of the supernatant was used for the IP. FMRP IP was performed using specific FMRP antibody (Ferrari et al, 2007) and Dynabeads Protein A immunoprecipitation kit (Invitrogen).

Samples were eluted in Laemmli buffer and supernatant were separated by SDS–PAGE electrophoresis and transferred to a PVDF membrane (Millipore). Membranes were incubated using specific antibodies for FMRP (Ferrari et al, 2007) (1:1000).

Immunoprecipitation followed by RT-qPCR

Cells were lysed in 100 or 250 mM NaCl, 50 mM Tris-HCl pH 7.4, 1% Triton X-100, 40 U ml^{-1} RNAse OUT (Invitrogen), 10 mg ml^{-1} PIC (Sigma). Lysis of mouse breast tumours was carried out on ice in 100 mM NaCl, 10 mM $MgCl_2$, 10 mM Tris-HCl pH 7.5, 1% Triton X-100, 1 mM DTT, 400 U ml^{-1} RNAse OUT (Invitrogen), 10 μl ml^{-1} PIC (Sigma), 5 mM β-glycerophosphate, 0,5 mM Na_3VO_4. After 5 min of incubation on ice, lysates were centrifuged for 5 min at 12,000g at 4°C and 500 μg of the supernatant was used for the IP. FMRP IP was performed using specific FMRP antibodies (Ferrari et al, 2007) or pre-immune rabbit IgGs as negative control and Dynabeads Protein A immunoprecipitation kit (Invitrogen).

RNA was extracted and reverse transcribed using RT² First-Stand cDNA Synthesis Kit (cat. 330401, SuperArray Bioscience-Qiagen) and applied to RT²Profiler™ PCR arrays (cat. PAMM-090F) as detailed by the manufacturer (Qiagen). Relative mRNA levels in immunoprecipitation samples were calculated as follows: $2^{-[\Delta Ct(specific\ IgGs) - \Delta Ct(CTR\ IgG)]} = 2^{-\Delta\Delta Ct}$, where ΔCt equals Ct (specific IgGs or CTR IgG) – Ct (*Input*). mRNAs with a fold change greater than 7,5 compared to CTR IgG were considered FMRP targets. The functional analyses of the proteins encoded by FMRP target mRNAs was generated through the use of IPA (Ingenuity® Systems, www.ingenuity.com)

IHC analysis on mouse tumours

The formalin-fixed paraffin embedded murine breast tumours and brains were stained (as defined in the manufaturer's staining protocol) on a Bond-maxTM fully auto- mated staining system (Leica Microsystems GmbH, Germany), using rabbit polyclonal antibodies for FMRP (Ferrari et al, 2007) (1:100), E-cadherin (1:100, BD) and goat antibodies for Vimentin (1:200, SIGMA) or deparaffinised, rehydrated and subjected to high temperature antigen retrieval in 1X Target Retrieval Solution (DAKO). Endogenous peroxidase activity was blocked by 3% H_2O_2. Subsequently, sections were incubated in 5% normal goat serum for FMRP or normal rabbit serum for E-cadherin and Vimentin. Mouse E-cadherin antibody (1:200, BD Pharmigen), mouse Vimentin antibody (1:200 SIGMA), rabbit affinity purified FMRP antibodies (Ferrari et al, 2007) (1:50) and affinity purified IgG from pre-immune serum, were used overnight at 4°C. Biotinylated goat anti-rabbit or rabbit anti-mouse were used. Samples were then incubated with the avidin-biotin or ABC peroxidase complexes (Vector Laboratiories). The immunoreaction product was revealed using aminoethylcarbazole (AEC) or 3-3' diaminobenzidine (DAB) as chromogenic substrates in presence of H_2O_2 (Biogenex). Control stainings were always performed omitting the primary antibody. Sections were counterstained in Mayer's acid hemalum

or Harris counterstaining and analysed. Mouse breast cancer samples were blindly evaluated by three independent observers using a light microscope. For each slice, a minimum of 10 fields was examined at 40X magnification. The Student's t-test was used. Statistical significance was set at $p < 0.05$.

RNA extraction and analysis

Total RNA from 4T1 CTR and *Fmr1* shRNA cells was isolated using TRIZOL Reagent (Invitrogen) and following the manufacturer's instruction. After *in vitro* retrotrascription a Real Time PCR was performed using an ABI 7300 Sequence Detector with dual-labeled TaqMan probes (Applied Biosystems). Mouse *Fmr1*, *Histone H3.3*, *E-cadherin* and *Vimentin* mRNAs were detected with Pre-Developed TaqMan gene expression assays Mm00484415_m1, Mm00787223_s1, Mm01247357_m1, Mm01333430_m1 respectively. mRNA levels were calculated as follows: $2^{-[\Delta Ct(\mathit{Fmr1}\ shRNA)-\Delta Ct(CTR\ shRNA)]} = 2^{-\Delta\Delta Ct}$, where ΔCt equals Ct(*E-cadherin*)-Ct(*Histone H3.3*). TaqMan Universal PCR Master Mix (ABI 4304437) was used.

Cycles: 2 min at 50° C and 10 min at 95° C, followed by 40 cycles of 15 sec at 95° C and 1 min at 60°.

Polysomes-mRNP analysis

4T1 cells were homogenized in 10mM Tris-HCl pH 7.5, 100mM NaCl, 10mM $MgCl_2$, 1% Triton-X100, 1mM dithiothreitol DTT, 40u/mL RNasin supplemented with 100 μg/ml cycloheximide. After 5 min of incubation on ice, the extract was centrifuged for 5 min at 12,000 g at 4°C. The supernatant was loaded onto a 15-50% (w/v) sucrose gradient and sedimented by centrifugation at 4°C for 110 min at 37,000 rpm in a Beckman SW41 rotor (Fullerton).

Each gradient was collected into 10 fractions followed by the addition of 1% SDS (final concentration), 40 pg of exogenous (*in vitro* transcribed) *BC200* RNA, 10μg glycogen, and proteinase K (100 μg/ml) and incubated for 30 min 37°C. The exogenous human *BC200* RNA (different sequences from BC1 in rodents) was used to monitor possible RNA loss during RNA phenol/chloroform extraction and precipitation from each fraction. RNAs were precipitated with 0.2M NaOAc and 0.7 vol of isopropanol. Pellets were then resuspended in 30 μl of ddH_2O. The RNA fractions 1-5 (polysomal fraction, P) and 6-10 (mRNP fraction, NP) were pooled and RNA quality/quantity was assessed by 1.8% agarose formaldehyde gel electrophoresis and spectrophotometry (ND-1000 spectrophotometer, Nanodrop Technology). mRNAs of interest

were quantified by RT-qPCR (see above), and the translational efficiency was calculated as follows: $2^{-[\Delta Ct(P)-\Delta Ct(NP)]} = 2^{-\Delta\Delta Ct}$, where ΔCt equals Ct (*specific* mRNAs) – Ct (*BC200* RNA).

Quantitative PCR was performed using Light Cycler 480 (Roche) and Sybr Green Master Mix (Roche) using the following primers:

1) *Histon* (mouse, *H3f3b*, PCR product = 77 bp)

fw: 5'-ACTGACTGTTCACAGACC-3'; rev: 5'-CCATCCCTTCTGCGTATTAG-3'

2) *E-cadherin* (mouse, *Cdh1*, PCR product = 63 bp)

fw: 5'-GTGACGCTGAAGTCCATGG-3'; rev: 5'-TTCAGAGGCAGGGTCGCG-3'

3) *Microtubule-associated protein 1B* (mouse, *Mtap1b*, PCR product=77 bp)

fw: 5'-TTC CAG GAC AAA AGA TTC TTC-3'; rev: 5'-GGC TTC ATC TGA AGG GTT GA-3'

4) *Caveolin2* (mouse, *Cav2*, PCR product=92 bp)

fw: 5'- GCT GTC TGC ACA TCT GGA TC-3'; rev: 5'-CGT CTG TCA CAC TCT TCC AT-3'

5) *Desmoplakin* (mouse, *Dsp*, PCR product=74 bp)

fw: 5'- ATA GCC ACA GGA GCA TCC AC-3'; rev: 5'- ACG CAG GTC TGC TTT GAT CT-3'

6) *Keratin 14* (mouse, *Krt14*, PCR product=85 bp)

fw: 5'-CAG CAA GAC AGA GGA GCT GA-3'; rev: 5'-CCG GAG CTC AGA AAT CTC AC-3'

7) *Microphthalmia-associated transcription factor* (mouse, *Mitf*, PCR product=83 bp)

fw: 5'-ACG TTA CCC GTC TCT GGA AA-3'; rev: 5'- CAG GAG TTG CTG ATG GTA AGG-3'

mRNA stability assay

4T1 CTR and *Fmr1* shRNA cells were treated from time 0 with Actinomycin D (1 mg ml^{-1}) for the indicates times. Cells were washed in D-PBS, the RNA extracted with Trizol and retrotrascribed with SuperScript III. Quantitative PCR was performed using Light Cycler 480 (Roche) and Sybr Green Master Mix (Roche) using the following primers:

1) *Histone* (mouse, *H3f3b*, PCR product = 77 bp)

fw: 5'-ACTGACTGTTCACAGACC-3'; rev: 5'-CCATCCCTTCTGCGTATTAG-3'

2) *Vimentin* (mouse, *Vim*, PCR product = 74 bp)

fw: 5'-CAAGGCCCGTGTCGAGGTGG-3'; rev: 5'-CCTCCTGCAATTTCTCTCGCAGCC-3'

3) *Fibronectin1* (mouse, *Fn1*, PCR product = 88 bp)

fw: 5'-GGAGCCTTCACACATCACCA-3'; rev: 5'-GTGGCCAGGAATGGTAGCTT-3'

4) *Jagged 1* (mouse, *Jag1*, PCR product = 85 bp)

fw: 5'-GTCCCAAGCATGGGTCTTGT-3'; rev: 5'-GGGATGCACTTGTCGCAGTA-3'

5) *Matrix metallopeptidase 9* (mouse, *Mmp9*, PCR product = 72 bp)

fw: 5'-CCTGGAACTCACACGACATCT-3'; rev: 5'-CACGCCAGAAGAATTTGCCAT-3'

6) *Serine (or cysteine) peptidase inhibitor, clade E, member 1* (mouse, *Serpine1*, PCR product = 104 bp)

fw: 5'-AATGACTGGGTGGAAAGGCAT-3'; rev: 5'-AAGTAGAGGGCATTCACCAGC-3'

7) *Epidermal growth factor receptor* (mouse, *Egfr*, PCR product = 105 bp)

fw: 5'-TGCCAATAATGTCTGCCACCT-3'; rev: 5'-TGCCATTGAACGTACCCAGAT-3'

Relative *mRNA* levels, normalized to *H3f3b*, were calculated as follows: $2^{-[\Delta Ct\ (Fmr1\ shRNA)\ -\ \Delta Ct(CTR\ shRNA)]} = 2^{-\Delta\Delta Ct}$, where ΔCt equals Ct(*specific mRNA*) – Ct (*H3f3b*).

Figure Legends

Supporting Information Fig S1. A. Two consecutive sections of a breast tumour TMA were stained with the same concentration of specific FMRP affinity purified antibodies (Ferrari et al, 2007) or the corresponding affinity-purified pre-immune IgGs. Shown are representative images. The breast tumour core was scored positive for FMRP using the specific antibody and negative with the pre-immune purified IgG. **B.** Western blot analysis of FMRP expression in WT and *Fmr1* KO mouse brain using specific Fmrp/FMRP antibodies (Ferrari et al, 2007). The signal appears clear in WT extracts and absent in *Fmr1* KO extracts, showing specificity. **C.** Immunohistochemistry detection of FMRP in formalin-fixed and paraffin-embedded mouse brains using in parallel the specific FMRP antibodies (affinity-purified IgGs) and the affinity-purified IgGs from the preimmune serum. The regions in the hippocampus known to contain FMRP are strongly highlighted showing the specificity of the anti-FMRP antibodies in immunohistochemistry. Scale bars: 500 μM (left panels) and 100μM (right panels). **D.** FMRP scoring on normal and tumour breast tissues on TMAs. Representative images of IHC for FMRP performed on different human breast tissues that show the range of FMRP staining (low = ≤1 to very high = >1). Scale bars = 100μm.

Supporting Information Fig S2. Kaplan-Meier curves of metastasis free probability. Left curve, EMC-344, MSK-99, NKI-295 datasets correlating *FMR1* mRNA levels with the probability of having metastasis to distal organs (lung excluded). *FMR1* low levels are indicated in red while high levels in blue. Middle curve: same as the left curve using only the NKI-295 node-negative data set. Right curve: same as left curve using only NKI-295 node positive dataset. pts = total

number of patients analysed (n = 569). *p*-values were calculated using the Log-rank test. n = number of patients; events = number of patients with distal metastasis. High levels of *FMR1 mRNA* do not correlate with an increased probability of lung metastasis to distant organs.

Supporting Information Fig S3. A, FMRP expression in mouse breast tumour cell lines (4T1, TS/A, n = 5, $p < 0.01$). FMRP levels were analysed by Western blot using specific FMRP antibodies (Ferrari et al, 2007) and normalised for Vinculin and Comassie staining (not shown). Quantification is reported in the histogram as ratio FMRP/Vinculin where FMRP levels in 4T1 cells were considered 100%. **B.** Silencing of *Fmr1* in 4T1 cells. 4T1 cells were transiently transfected with five different shRNAs as well as with a scrambled shRNA (see Methods). Fmrp levels were detected by Western blot, normalised to Vinculin and values reported in the histogram as ratio Fmrp /Vinculin where Fmrp levels in CTR cells were considered 100%. **C.** 4T1 cells silenced for FMRP. Cells were stably tranduced with different combination of three shRNAs against the *Fmr1* gene. Fmrp levels were detected by Western blot and normalised to Vinculin or total proteins (Coomassie staining, data not shown). Fmrp levels are expressed as a ratio to control cells (100%). **D.** Same as panel (C) for the TS/A cell line. **E.** Primary tumour growth after orthotopic injection of control (CTR) and *Fmr1* silenced 4T1 cells in WT syngenic mice (n = 12, $p = 0.09$, two-way ANOVA applying the Bonferroni correction). The graph represents tumour volume as a function of time after the injection. **F.** Primary tumour growth after orthotopic injection of control (CTR) *and Fmr1* silenced TS/A cells in WT syngenic mice (n = 13, $p = 0.23$, two-way ANOVA applying Bonferroni correction). The graph represents tumour volume as a function of time after the injection.

Supporting Information Fig S4. A. IHC analysis of Fmrp expression in primary tumours generated by orthotpic injection of CTR or *Fmr1* shRNA TS/A cells. Shown is an overview (left and right panels) or a detail (middle panel) of FMRP-stained primary tumours created after injection of control (left and middle panels) or *Fmr1*-silenced TS/A cells (right panel). The tumour margin is marked by a black arrow and also shown in the enlarged detail. **B.** *Fmr1* mRNA expression levels in tumours generated with shRNA vs *Fmr1* shRNA, (n = 5, $p < 0.05$, Student's t test). **C**. FMRP levels in normal and primary tumour tissues by Western blotting analysis. Lanes 1-3, protein extracts from breast tissues (mammary fat pad); lanes 4-11, 8 different murine breast tumours. FMRP levels were analysed by Western blot using specific FMRP antibodies (Ferrari et al, 2007) (left upper panel), the signal was normalised for Coomassie staining of the membrane (left lower panel). The right panel reports the quantification of the signals intensity ($p < 0.01$,

Student's t test). **D**. Lung metastasis generated after orthotopic injection of control or *Fmr1* silenced TS/A cells were analyzed for FMRP expression levels. Black arrows on left panels point to the metastasis, one of them is enlarged on the right panel. Scale bars: 200 μm (left panels) and 20 μm (right panels).

Supporting Information Fig S5. A. Fmrp and GFP expression in 4T1 cells. 4T1 cells were transiently transfected using a recombinant plasmid containing the *Fmr1* shRNA (#3 or #4) under the U6 promoter and the GFP in a separate transcription unit. Fmrp was detected by immunofluorescence. Scale bars: 20 μm. **B.** Cancer cell survival in the bloodstream. Mice were injected intravenously (I.V.) with *Fmr1* silenced and control 4T1 cells, blood was collected 6 and 24 hours after the injection (n = 5), and analysed as in panel B. **C.** Lung metastasis formation in I.V. injected mice (n = 5).

Supporting Information Fig S6. A. 4T1 cell morphology after Ca^{2+} deprivation. Upper panels: phase-contrast images of 4T1 cells expressing CTR shRNA and CTR vector after 18 min of EDTA treatment, lower panels: 4T1 cells expressing *Fmr1* shRNA and *GFP-FMR1*. All insets show time = 0. Scale bar: 50 μm. **B.** Cell area in CTR and *Fmr1* shRNA cells (n = 18.590, $p < 0.01$) **C.** Transendhotelial migration assay of CTR shRNA and *Fmr1* shRNA 4T1 cells (n = 5, $p < 0{,}01$). Histograms show the quantification of migrating cells. **D.** 3D tumour cell spheroids invasion assay. 4T1 cells, stably transduced with CTR shRNA and *Fmr1* shRNA #4/5 and #3/5 (unpublished observations), were embedded in a collagene type I gel and imaged after 24hrs. White dotted line indicates the spheroid body. Left panels indicate the quantification of the different parameters analyzed (n= 15 per condition, $p < 0.001$, $p < 0.001$, Student's t-test). Scale bar = 200 μm.

Supporting Information Fig S7. FMRP immunoprecipitation from 4T1 cells with specific FMRP antibodies (Ferrari et al, 2007) and affinity purified pre-immune IgGs visualized by Western blotting.

Supporting Information Fig. S8. Immunohistochemistry of FMRP, E-cadherin and Vimentin performed on human non-metastatic (BC) and metastatic (mBC) breast cancer (upper panels). Lower panel shows a correlation between FMRP, E-cadherin and Vimentin in BC and mBC (n = 14).

Supporting Information Fig. S9. mRNA stability assay in 4T1 CTR and *Fmr1* shRNA cells. RNA was isolated at the indicated time points after Actinomycin D treatment and the stability of *Fn1*, *Jag1*, *Mmp9*, *Serpine1* and *Egfr* mRNAs was analyzed by RT-qPCR (n = 5, * p <0.05, ** p < 0.01).

Supporting Information Fig. S10. Translational efficiency analysis in 4T1 CTR and Fmr1 shRNA cells. Quantification of the translational efficiency of Histone *H3.3, Mtap1b, Cav2, Dsp, Krt14* and *Mitf* mRNAs reported as ratio of P over NP ($2^{-[\Delta Ct(P) - \Delta Ct(NP)]}$) (n = 4, * $p < 0.05$, ** $p <$ 0.01).

Supporting Information Fig S11. A. *E-cadherin* and *Vimentin* mRNA levels in 4T1 CTR and *Fmr1* siRNA (Ambion (ID #10919) cells detected by RT-qPCR (n = 3, $p < 0.05$). **B.** Translational efficiency analysis in 4T1 CTR and *Fmr1* siRNA cells. Upper panel, polysome-mRNPs distribution on a sucrose gradient. Low left panel, fractions 1 to 5 corresponding to translating polysomes (P) and fractions 6 to 10 corresponding to silent mRNPs (NP) were pooled. Low right panel, quantification of the translational efficiency of *Histone H3.3*, *E-cadherin* and *Vimentin* mRNAs reported as ratio of P over NP ($2^{-[\Delta Ct(P) - \Delta Ct(NP)]}$) (n = 3, $p < 0.01$).

Supporting Information Fig S1

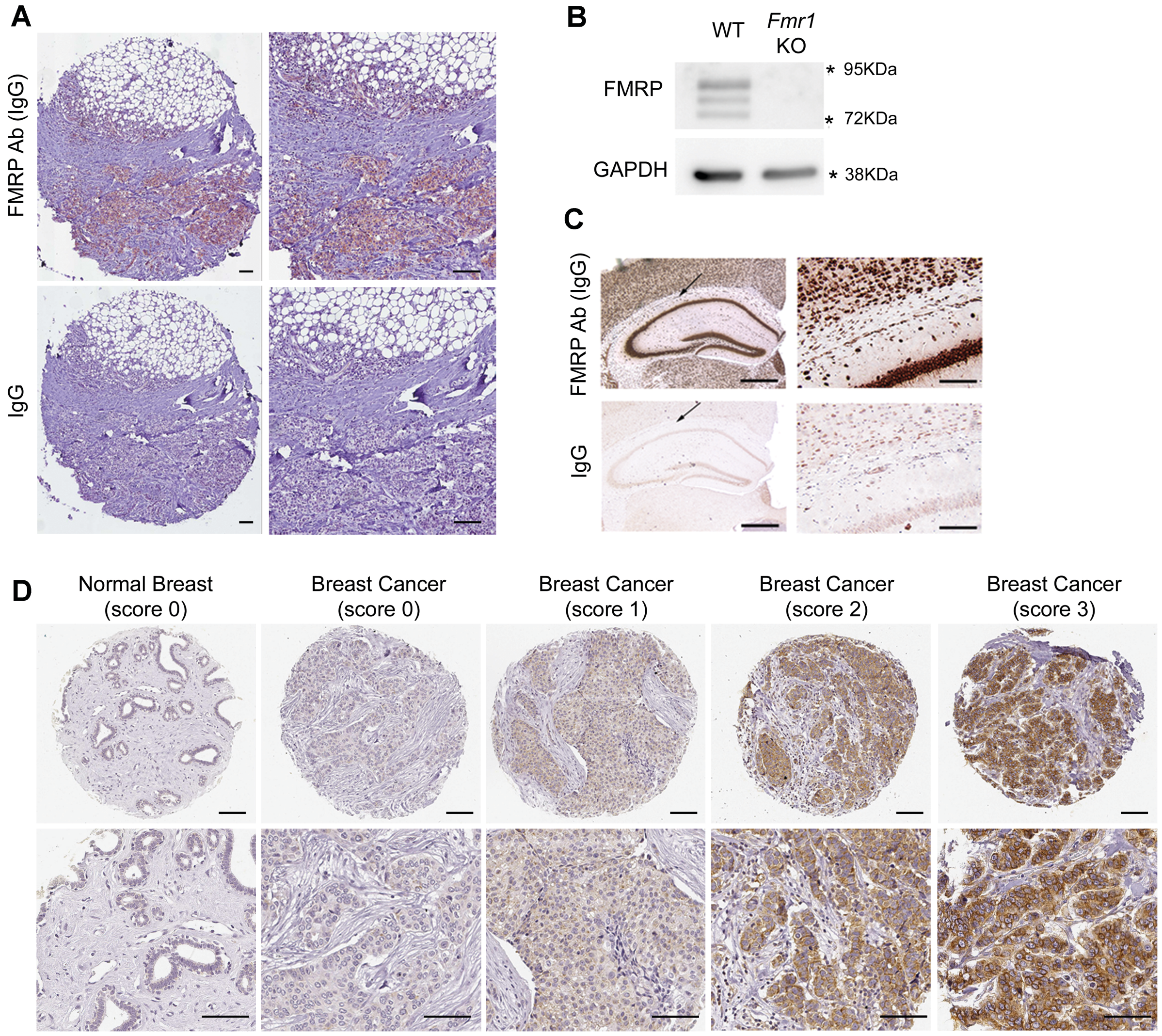

Supporting Information Fig S2

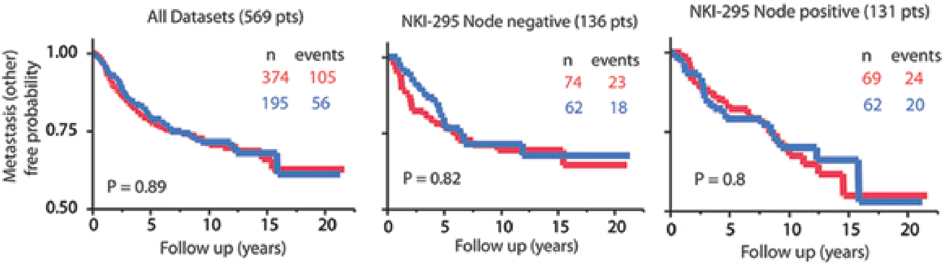

Supporting Information Fig S3

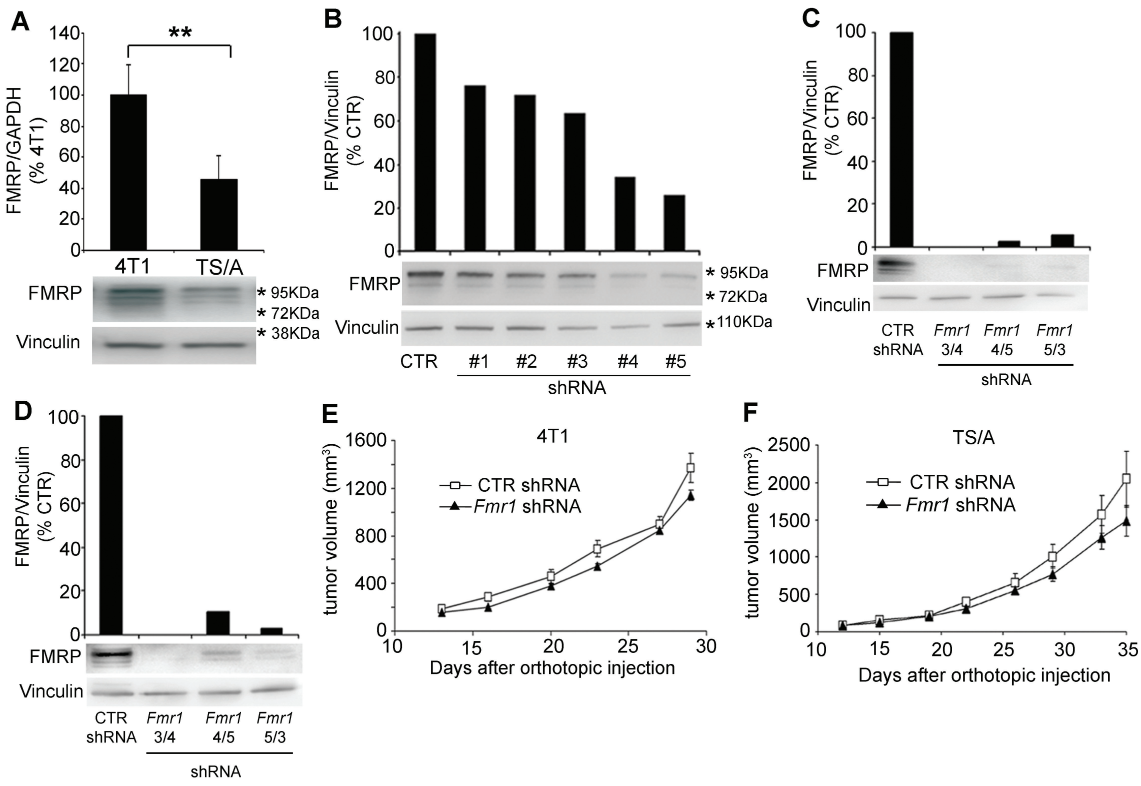

Supporting Information Fig S4

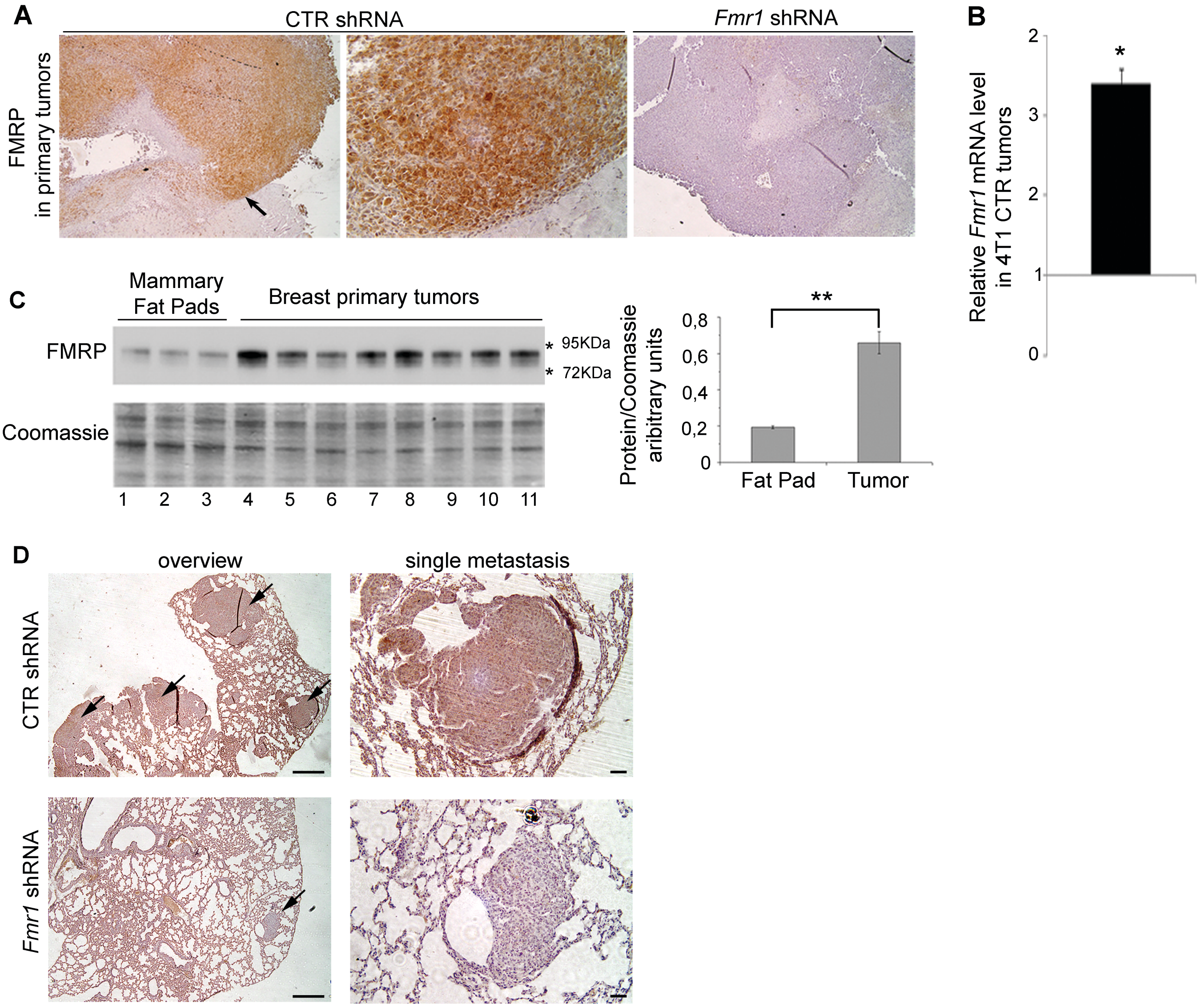

Supporting Information Fig S5

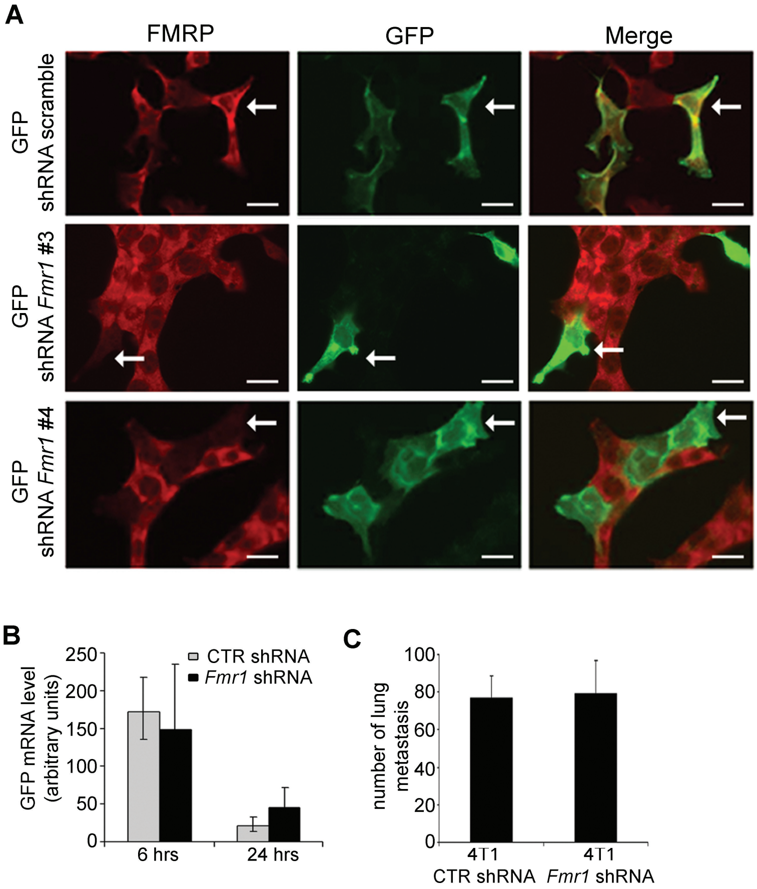

Supporting Information Fig S6

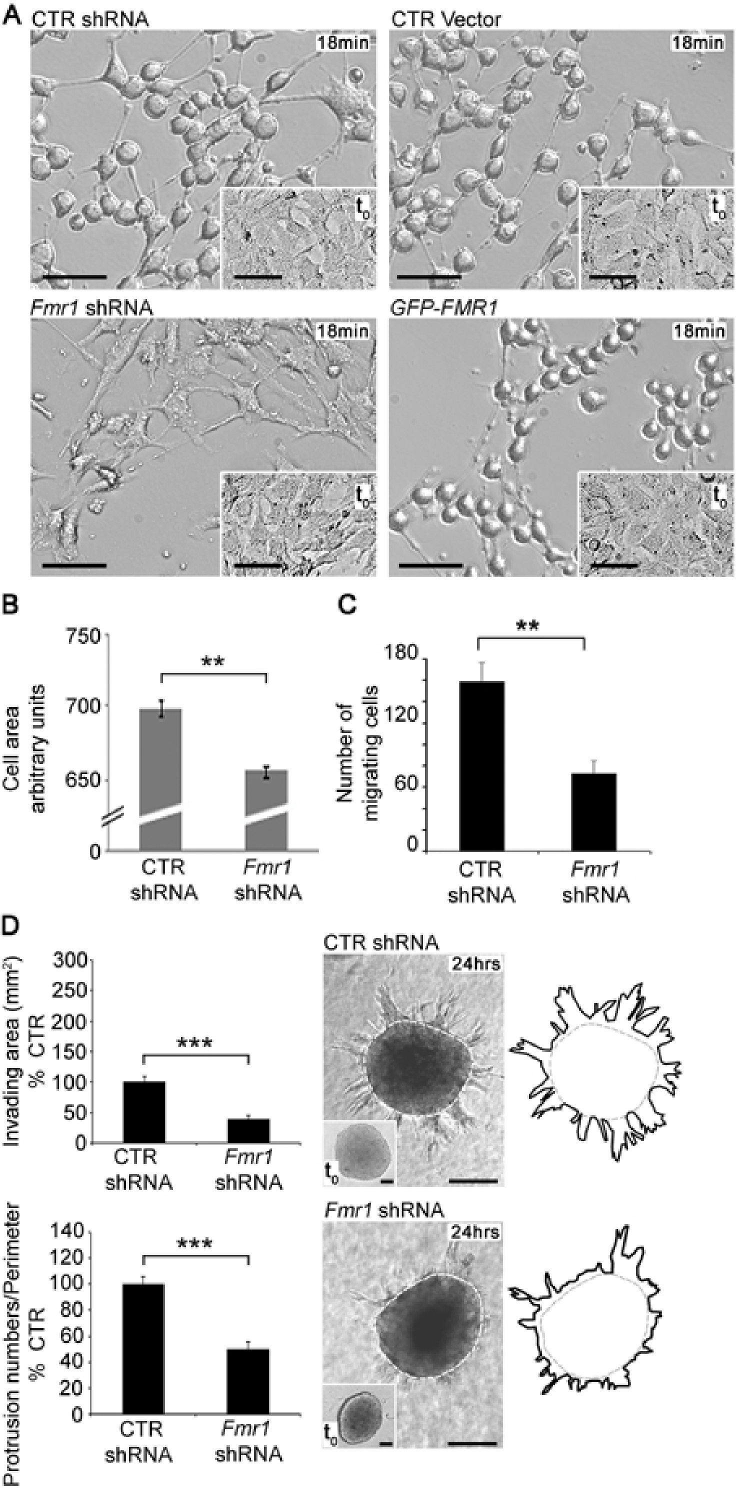

Supporting Information Fig S7

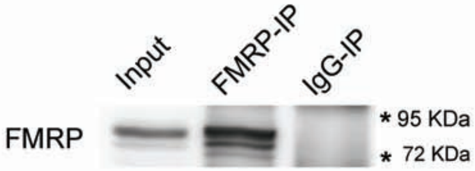

Supporting Information Fig S8

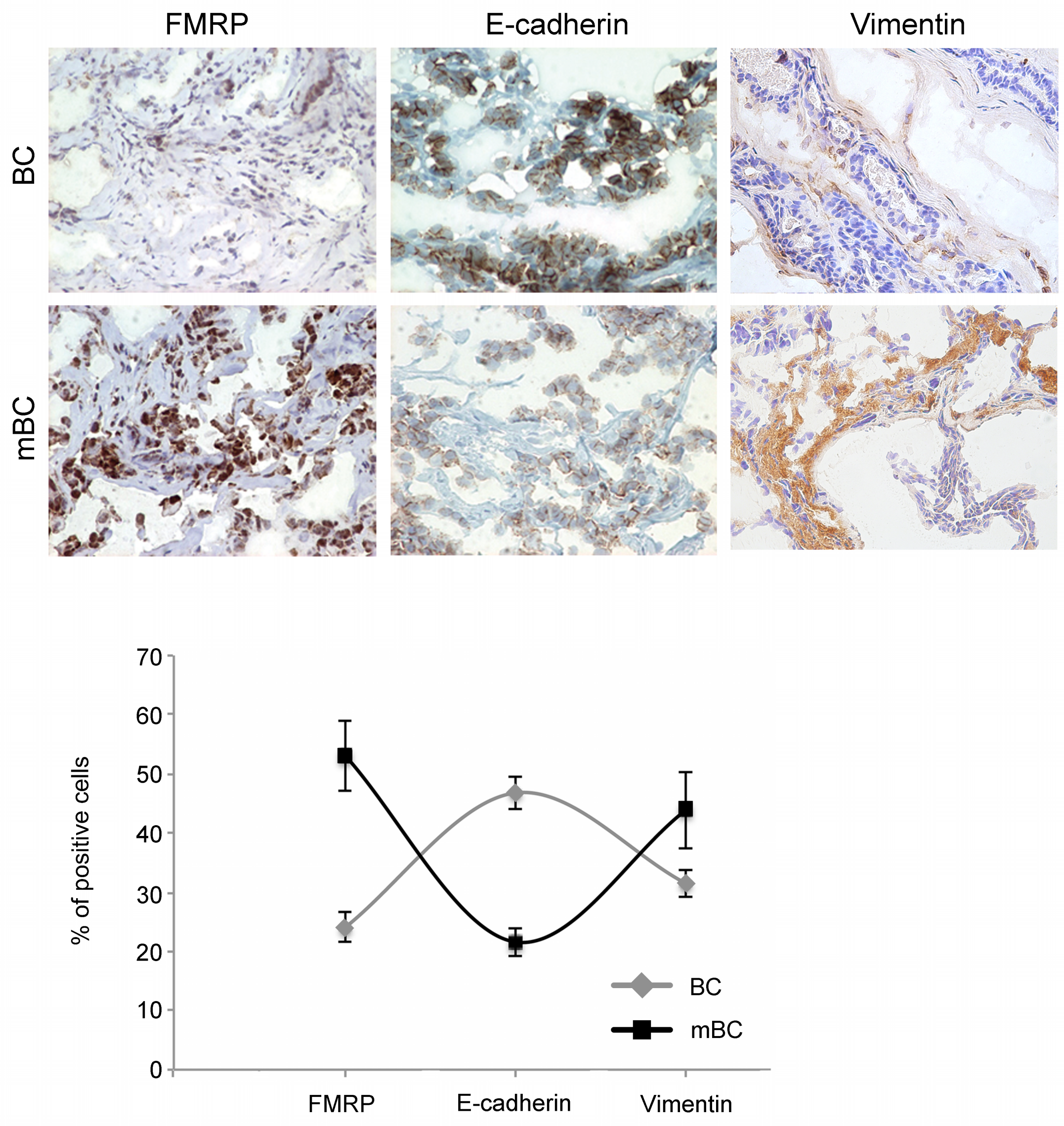

Supporting Information Fig S9

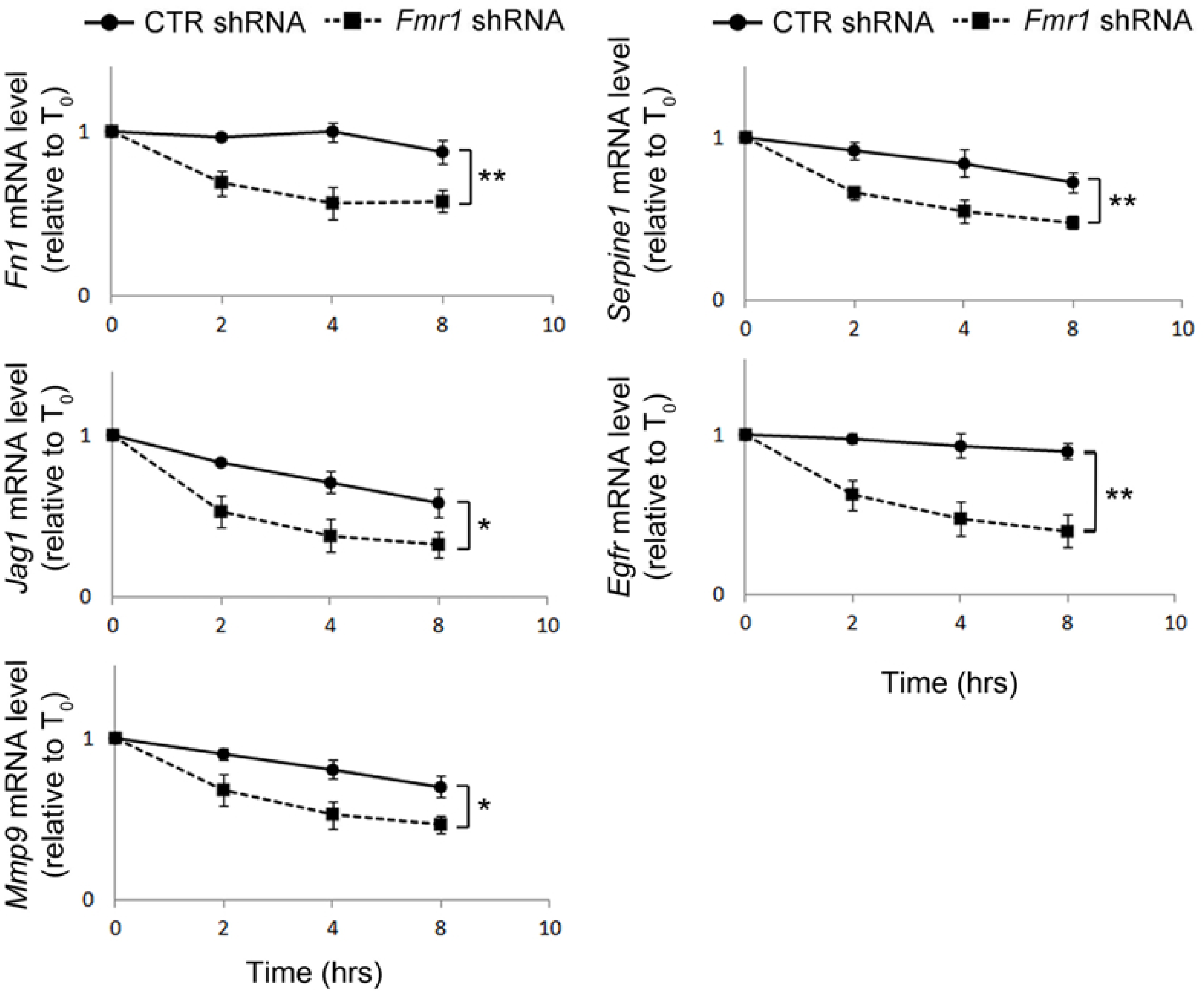

Supporting Information Fig S10

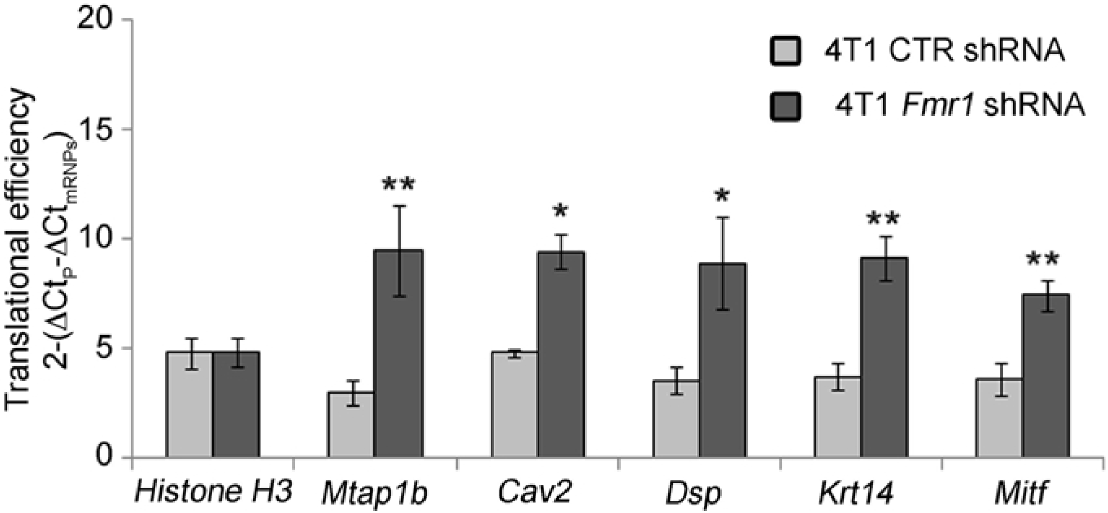

Supporting Information Fig S11

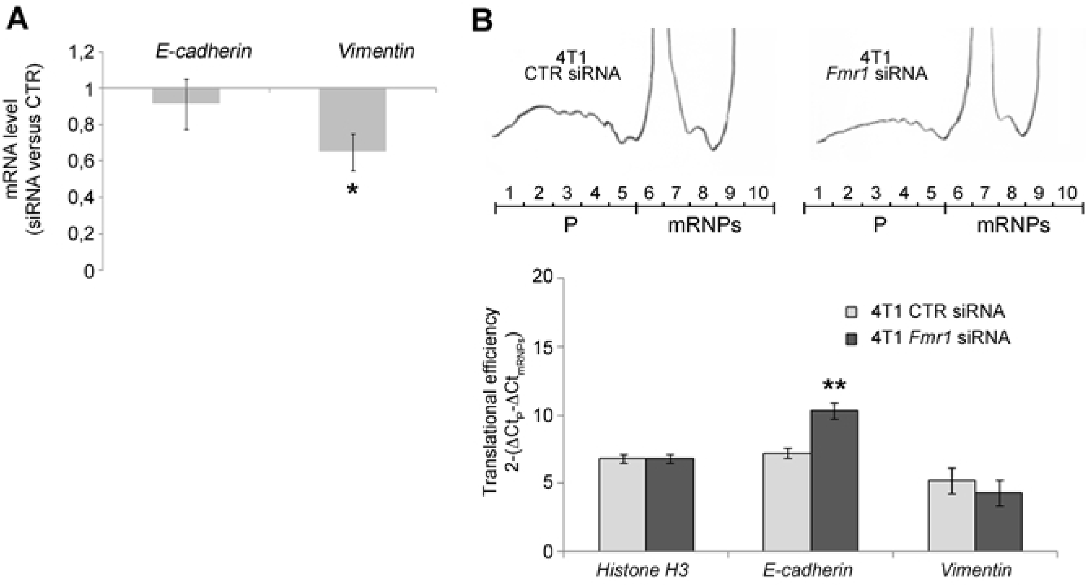

Supporting Information Table S1

A TMAs used to detect FMRP expression

Organ	TMA-1 (T/N)[A]	TMA-2 (T/N)	TMA-3 (T/N)	TMA-4 (T/N)
**Breast**	19/17*		28/14*	
**Colo-rectum**	19/7			28/28
**Lung**	20/5	28/28		
**Prostate**				20/20
**Bladder**				12/12

B FMRP expression on the TMA

Organ	Type	Positive (*n*)	Positive (%)
**Lung**	N[B]	2 (32)	7.1
	T[C]	4 (45)	8.9
**Prostate**	N	0 (20)	0
	T	1 (20)	5
**Colon**	N	2 (26)	7.7
	T	18 (36)	50
**Bladder**	N	1 (8)	12.5
	T	5 (13)	38.5

[A]Number of tumor cases/normal matched samples. *normal samples fibroadenomas; [B]Normal tissue; [C] tumor tissue; in parenthesis percentage of FMRP positive tumor.

Supporting Information Table S2

Clinical and pathological informations of the consecutive cohort of breast cancer patients (IFOM-Milan).

Parameter	Group	CONSECUTIVE COHORT (n = 477)	
		**n**[A]	**%**[B]
**Age**	*< 50*	120	25.2
	≥ 50	357	74.8
**Histotype**	*Ductal*	445	93.3
	Lobular	32	6.7
	Other	-	-
***pT**[C]	*1*	123	25.8
	2	353	74.2
	3	-	-
	4	-	-
***Nodal Status**[D]	*Neg*	303	63.7
	Pos	173	36.3
***†GRADE**[E]	*1*	145	30.7
	2	229	48.5
	3	98	20.8
***ER**[F]	*Neg*	44	9.2
	Pos	432	90.8
***PgR**[G]	*Neg*	145	30.5
	Pos	331	69.5
***Ki-67**[H]	*Neg*	253	53.3
	Pos	222	46.7
***ErbB2**[I]	*0*	364	78.5
	1	59	12.6
	2	12	2.6
	3	29	6.3
***ALL EVENTS (ANY)**[J]	*event*	32	6.7
	no event	444	93.3
***DISTANT EVENTS**[K]	*event*	20	4.2
	no event	456	95.8

[A]Number of patients; [B]percentage of patients in the categories; [C]primary tumour stage; [D]Lymph nodes involvement; [E]tumor grade; [F]estrogen receptor status; [G]progesterone receptor status; [H]proliferation index; [I]Human Epidermal growth factor Receptor 2 status; [J]loco-regional relapse, distant metastasis or controlateral breast cancer; [K]distant metastasis. *Not all the parameters were available for all the patients analysed.

Supporting Information Table S3

Clinical and pathological informations of the breast cancer samples (UZLeuven).

Parameter	Group	CONSECUTIVE COHORT (n = 12) n[A]	%[B]
**Age**	*< 50*	4	33.3
	≥ 50	8	66.7
**Histotype**	*Ductal*	12	100
	Lobular	-	-
	Other	-	-
**pT[C]**	*1*	4	33.3
	2	5	41.7
	3	3	25
	4	-	-
**Nodal Status[D]**	*Neg*	6	50
	Pos	6	50
**†GRADE[E]**	*1*	1	8.3
	2	5	41.7
	3	6	50
**ER[F]**	*Neg*	2	16.7
	Pos	10	83.3
**PgR[G]**	*Neg*	6	50
	Pos	6	50
***ErbB2[H]**	*Neg*	2	16.7
	Pos	7	58.3
**ALL EVENTS (ANY)[I]**	*event*	12	100
	no event	-	-
**DISTANT EVENTS[J]**	*event*	12	100
	no event	-	-

[A]Number of patients; [B]percentage of patients in the categories; [C]primary tumour stage; [D]Lymph nodes involvement; [E]tumor grade; [F]estrogen receptor status; [G]progesterone receptor status; [H]Human Epidermal growth factor Receptor 2 status; [I]loco-regional relapse, distant metastasis or controlateral breast cancer; [J]distant metastasis. *Not all the parameters were available for all the patients analysed.

Supporting Information Table S4

A Cancer occurring in the Cohort of 241 women with *FMR1* pre-mutation and full-mutation.

Carrier status (all females)	pts (*n*)	age (range)	age (median)	breast cancers	other cancers	pts (died) from cancer
**pre-mutation**	164	11-88	52	1 ductal carcinoma (72 yrs)	1 granulosa cell ovary (63 yrs)<br>1 lymphoma malignant (44 yrs)<br>1 pseudomyxoma peritonei (73 yrs)	1 met Breast Cancer<br>1 lymphoma
**full mutation**	35	9-87	46		1 liver (50 yrs)	1 liver cancer
**unaffected full mutation**	42	8-68	33	1 ductal carcinoma (47 yrs)	1 lung (63 yrs)<br>1 gastric cancer mets to ovary (45 yrs)	1 lung cancer<br>1 gastric cancer
**Total**	241			2	6	

B Cancer incidence in the Cohort of 199 women with *FMR1* pre-mutation and full-mutation

Carrier status (all females)	Type of cancer	Cases observed[A]	Cases predicted[B]	*p* value
**Pre-mutation + Full mutation**	breast cancer	1	5.79	0.02
	all cancers	5	15.93	0.001

[A]Individuals with the *FMR1* pre and full mutation; [B]UK population

Supporting Information Table S5

FMRP target mRNAs in breast cancer cells.

Gene	RefSeq	Official Name	Fold Change
Ahnak	NM_001039959	AHNAK nucleoprotein (desmoyokin)	65,07
Akt1	NM_009652	Thymoma viral proto-oncogene 1	44,61
Cav2	NM_016900	Caveolin 2	7,70
Cdh1	NM_009864	Cadherin 1	15,63
Ctnnb1	NM_007614	Catenin (cadherin associated protein), beta 1	22,29
Dsp	NM_023842	Desmoplakin	49,48
Egfr	NM_007912	Epidermal growth factor receptor	28,84
Erbb3	NM_010153	V-erb-b2 erythroblastic leukemia viral oncogene homolog 3 (avian)	85,25
Esr1	NM_007956	Estrogen receptor 1 (alpha)	35,04
Fn1	NM_010233	Fibronectin 1	23,15
Foxc2	NM_013519	Forkhead box C2	16,23
Igfbp4	NM_010517	Insulin-like growth factor binding protein 4	9,32
Ilk	NM_010562	Integrin linked kinase	25,39
Itga5	NM_010577	Integrin alpha 5 (fibronectin receptor alpha)	38,03
Itgb1	NM_010578	Integrin beta 1 (fibronectin receptor beta)	16,44
Jag1	NM_013822	Jagged 1	16,93
Krt14	NM_016958	Keratin 14	16,42
Mitf	NM_008601	Microphthalmia-associated transcription factor	18,51
Mmp3	NM_010809	Matrix metallopeptidase 3	15,47
Mmp9	NM_013599	Matrix metallopeptidase 9	22,42
Msn	NM_010833	Moesin	8,26
Mtap1b	NM_008634	Microtubule-associated protein 1B	28,74
Notch1	NM_008714	Notch gene homolog 1 (Drosophila)	32,23
Ocln	NM_008756	Occludin	18,27
Pppde2	NM_134095	PPPDE peptidase domain containing 2	15,63
Ptk2	NM_007982	PTK2 protein tyrosine kinase 2	60,02
Ptp4a1	NM_011200	Protein tyrosine phosphatase 4a1	11,20
Serpine1	NM_008871	Serine (or cysteine) peptidase inhibitor, clade E, member 1	19,51
Smad2	NM_010754	MAD homolog 2 (Drosophila)	42,12
Sparc	NM_009242	Secreted acidic cysteine rich glycoprotein	12,61
Spp1	NM_009263	Secreted phosphoprotein 1	9,57
Stat3	NM_011486	Signal transducer and activator of transcription 3	17,25
Steap1	NM_027399	Six transmembrane epithelial antigen of the prostate 1	8,43
Tgfb1	NM_011577	Transforming growth factor, beta 1	17,01
Tgfb2	NM_009367	Transforming growth factor, beta 2	29,18
Tgfb3	NM_009368	Transforming growth factor, beta 3	21,76
Tmeff1	NM_021436	Transmembrane protein with EGF-like and two follistatin-like domains 1	12,51
Tmem132a	NM_133804	Transmembrane protein 132A	54,09
Twist1	NM_011658	Twist homolog 1 (Drosophila)	14,94
Vim	NM_011701	Vimentin	7,82
Vps13a	NM_173028	Vacuolar protein sorting 13A (yeast)	78,89
Zeb2	NM_015753	Zinc finger E-box binding homeobox 2	30,31

Supporting Information Table S6

Clinical and pathological informations of the breast cancer samples (San Giovanni Rotondo Hospital).

Parameter	Group	CONSECUTIVE COHORT (n = 18)	
		n[A]	%[B]
**Age**	*< 50*	5	27.8
	≥ 50	13	72.2
**Histotype**	*Ductal*	18	100
	Lobular	-	-
	Other	-	-
**pT**[C]	*1*	4	22.2
	2	8	44.4
	3	1	5.6
	4	5	27.8
**Nodal Status**[D]	*Neg*	6	33.3
	Pos	12	66.7
***GRADE**[E]	*1*	2	11.1
	2	9	50
	3	6	33.3
***ER**[F]	*Neg*	6	33.3
	Pos	11	61.1
***PgR**[G]	*Neg*	10	55.6
	Pos	7	38.9
***Ki-67**[H]	*Neg*	2	11.1
	Pos	9	50
***ErbB2**[I]	*Neg*	11	61.1
	Pos	6	33.3
**ALL EVENTS (ANY)**[J]	*event*	12	66.7
	no event	6	33.3
**DISTANT EVENTS**[K]	*event*	2	11.1
	no event	16	88.9

[A]Number of patients; [B]percentage of patients in the categories; [C]primary tumour stage; [D]Lymph nodes involvement; [E]tumor grade; [F]estrogen receptor status; [G]progesterone receptor status; [H]proliferation index; [I]Human Epidermal growth factor Receptor 2 status; [J]loco-regional relapse, distant metastasis or controlateral breast cancer; [K]distant metastasis. *Not all the parameters were available for all the patients analysed.

REFERENCES

Capra M, Nuciforo PG, Confalonieri S, Quarto M, Bianchi M, Nebuloni M, Boldorini R, Pallotti F, Viale G, Gishizky ML et al (2006) Frequent alterations in the expression of serine/threonine kinases in human cancers. Cancer Res 66**:** 8147-8154

Chen C, Okayama H (1987) High-efficiency transformation of mammalian cells by plasmid DNA. Mol Cell Biol 7**:** 2745-2752

Confalonieri S, Quarto M, Goisis G, Nuciforo P, Donzelli M, Jodice G, Pelosi G, Viale G, Pece S, Di Fiore PP (2009) Alterations of ubiquitin ligases in human cancer and their association with the natural history of the tumor. Oncogene 28**:** 2959-2968

Del Duca D, Werbowetski T, Del Maestro RF (2004) Spheroid preparation from hanging drops: characterization of a model of brain tumor invasion. J Neurooncol 67**:** 295-303

Ferrari F, Mercaldo V, Piccoli G, Sala C, Cannata S, Achsel T, Bagni C (2007) The fragile X mental retardation protein-RNP granules show an mGluR-dependent localization in the post-synaptic spines. Mol Cell Neurosci 34**:** 343-354

Freeley M, Bakos G, Davies A, Kelleher D, Long A, Dunican DJ (2010) A high-content analysis toolbox permits dissection of diverse signaling pathways for T lymphocyte polarization. J Biomol Screen 15**:** 541-555

Hattermann K, Held-Feindt J, Mentlein R (2011) Spheroid confrontation assay: a simple method to monitor the three-dimensional migration of different cell types in vitro. Ann Anat 193**:** 181-184

Kononen J, Bubendorf L, Kallioniemi A, Barlund M, Schraml P, Leighton S, Torhorst J, Mihatsch MJ, Sauter G, Kallioniemi OP (1998) Tissue microarrays for high-throughput molecular profiling of tumor specimens. Nat Med 4**:** 844-847

Naldini L, Blomer U, Gallay P, Ory D, Mulligan R, Gage FH, Verma IM, Trono D (1996) In vivo gene delivery and stable transduction of nondividing cells by a lentiviral vector. Science 272**:** 263-267

Veronesi U, Paganelli G, Viale G, Luini A, Zurrida S, Galimberti V, Intra M, Veronesi P, Robertson C, Maisonneuve P et al (2003) A randomized comparison of sentinel-node biopsy with routine axillary dissection in breast cancer. N Engl J Med 349**:** 546-553
